# Data mining of PubChem bioassay records reveals diverse OXPHOS inhibitory chemotypes as potential therapeutic agents against ovarian cancer

**DOI:** 10.1186/s13321-024-00906-0

**Published:** 2024-10-07

**Authors:** Sejal Sharma, Liping Feng, Nicha Boonpattrawong, Arvinder Kapur, Lisa Barroilhet, Manish S. Patankar, Spencer S. Ericksen

**Affiliations:** 1 University of Wisconsin-Madison, Department of Obstetrics and Gynecology, Madison, WI 53705 USA; 2 Department of Obstetrics and Gynecology, Qilu Hospital of Shandong University, Jinan, Shandong 250012 People’s Republic of China; 3 University of Wisconsin-Madison, UW-Carbone Cancer Center, Drug Development Core, Small Molecule Screening Facility, Wisconsin Institutes for Medical Research, 1111 Highland Avenue, Madison, WI 53705 USA

**Keywords:** Data mining, PubChem, Oxidative phosphorylation, Electron transport chain, Ovarian cancer, Cheminformatics, Bioenergetics assays, Cancer therapeutics

## Abstract

Focused screening on target-prioritized compound sets can be an efficient alternative to high throughput screening (HTS). For most biomolecular targets, compound prioritization models depend on prior screening data or a target structure. For phenotypic or multi-protein pathway targets, it may not be clear which public assay records provide relevant data. The question also arises as to whether data collected from disparate assays might be usefully consolidated. Here, we report on the development and application of a data mining pipeline to examine these issues. To illustrate, we focus on identifying inhibitors of oxidative phosphorylation, a druggable metabolic process in epithelial ovarian tumors. The pipeline compiled 8415 available OXPHOS-related bioassays in the PubChem data repository involving 312,093 unique compound records. Application of PubChem assay activity annotations, PAINS (Pan Assay Interference Compounds), and Lipinski-like bioavailability filters yields 1852 putative OXPHOS-active compounds that fall into 464 clusters. These chemotypes are diverse but have relatively high hydrophobicity and molecular weight but lower complexity and drug-likeness. These chemotypes show a high abundance of bicyclic ring systems and oxygen containing functional groups including ketones, allylic oxides (alpha/beta unsaturated carbonyls), hydroxyl groups, and ethers. In contrast, amide and primary amine functional groups have a notably lower than random prevalence. UMAP representation of the chemical space shows strong divergence in the regions occupied by OXPHOS-inactive and -active compounds. Of the six compounds selected for biological testing, 4 showed statistically significant inhibition of electron transport in bioenergetics assays. Two of these four compounds, lacidipine and esbiothrin, increased in intracellular oxygen radicals (a major hallmark of most OXPHOS inhibitors) and decreased the viability of two ovarian cancer cell lines, ID8 and OVCAR5. Finally, data from the pipeline were used to train random forest and support vector classifiers that effectively prioritized OXPHOS inhibitory compounds within a held-out test set (ROCAUC 0.962 and 0.927, respectively) and on another set containing 44 documented OXPHOS inhibitors outside of the training set (ROCAUC 0.900 and 0.823). This prototype pipeline is extensible and could be adapted for focus screening on other phenotypic targets for which sufficient public data are available.

**Scientific contribution**

Here, we describe and apply an assay data mining pipeline to compile, process, filter, and mine public bioassay data. We believe the procedure may be more broadly applied to guide compound selection in early-stage hit finding on novel multi-protein mechanistic or phenotypic targets. To demonstrate the utility of our approach, we apply a data mining strategy on a large set of public assay data to find drug-like molecules that inhibit oxidative phosphorylation (OXPHOS) as candidates for ovarian cancer therapies.

**Graphical Abstract:**

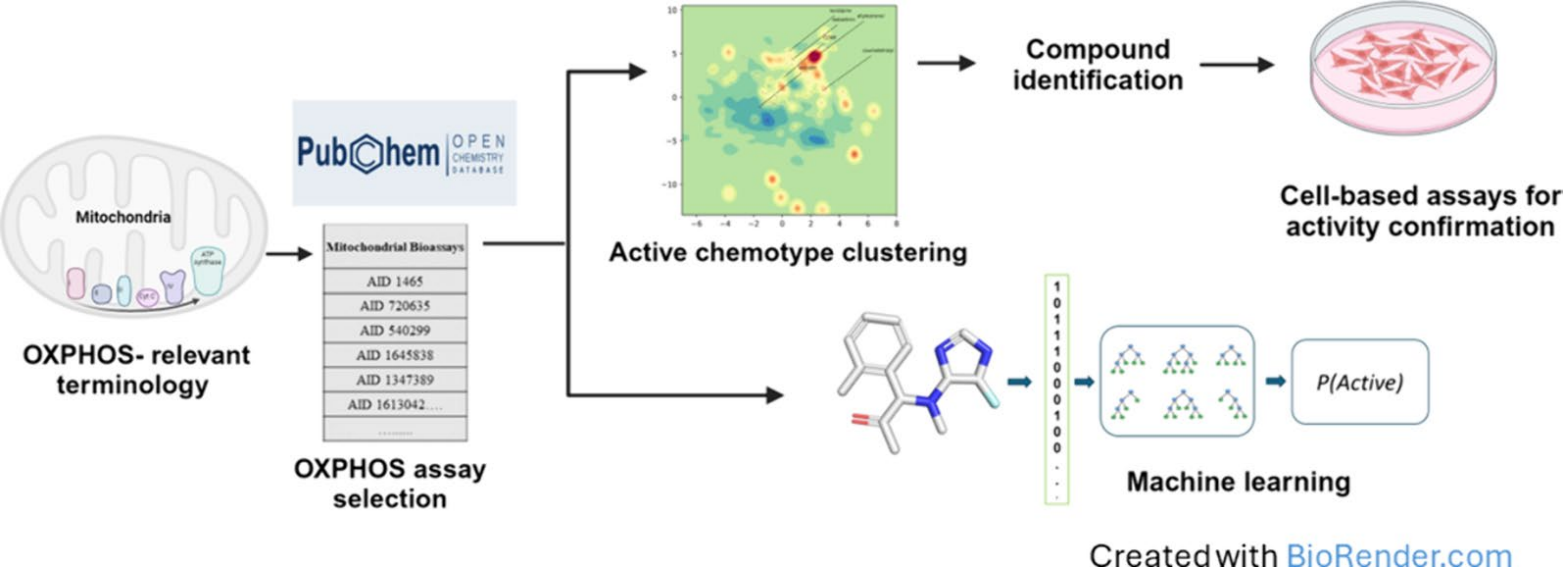

**Supplementary Information:**

The online version contains supplementary material available at 10.1186/s13321-024-00906-0.

## Introduction

The search for novel therapeutic compounds that target specific biological pathways remains a major undertaking in our quest for finding cures for many maladies. High throughput screening (HTS) remains a prominent approach for finding active compounds as starting points for probe or drug development. Given the resource commitment with HTS, investigators studying disease mechanisms have turned to smaller, focused screens using molecules prioritized by virtual screening (VS) models [1–3]. Both structure-based and ligand-based VS (LBVS) models continue to scale with rapidly expanding synthesize-on-demand virtual chemical libraries, now exceeding 40 billion molecules [4]. The LBVS models, however, have a distinct advantage in that they are broadly applicable to predicting any assay read-out including pathway or phenotypic end points where a specific protein target is unknown. The key limitation of such models, however, is that they may only be applied to targets for which ample molecule screening data are available for model training. For novel targets where such data are lacking, HTS assay data involving related protein targets might be used for training in a transfer learning-styled approach [5–7]. However, in such cases, it may not be clear which screening data sets should be included or whether data from a collection of varied assays can be effectively integrated for SAR-based data mining and model training. Here as a case study, we examine the utility in pooling disparate assay data sets associated with a multi-protein, anti-cancer target pathway to prioritize inhibitory chemical classes/scaffolds (chemotypes) and train LBVS models to guide focused screening.

There exists a plethora of pharmacological data on drug-like molecules in published literature from investigators around the world and through the last several decades (in some cases even the last century). With the establishment of PubChem [8], ChEMBL [9], BindingDB [10], and similar databases, vast chemical screening data have been collated and shared publicly. For example, PubChem, at the time of this writing, contains over 116 million compound records (CIDs) associated with > 305 million bioassay results. We propose that careful selection and mining of such data can facilitate identification of drug-like molecules to test in focused screens. Here, we describe and apply an assay data mining pipeline to compile, process, filter, and mine public bioassay data. We believe the procedure may be more broadly applied to guide compound selection in early-stage hit finding on novel multi-protein mechanistic or phenotypic targets. To demonstrate the utility of our approach, we apply a data mining strategy on a large set of public assay data sets to find drug-like molecules that inhibit oxidative phosphorylation (OXPHOS). In the OXPHOS pathway, five multimeric protein complexes of the inner mitochondrial membrane cooperate to synthesize ATP. Inhibition of OXPHOS often results in a rapid increase in intracellular oxygen radicals, which in cancer cells cause DNA damage and cell death. Major efforts are underway to develop novel OXPHOS inhibitors that are safe and effective for treatment of ovarian and other malignancies [11–13]. The multiple proteins required for the functioning of OXPHOS provide an excellent opportunity to test the utility of our data mining approach to identify new small molecule anti-cancer drugs and hence is the focus of the work presented here. While some OXPHOS inhibitors such as atovaquone have proven to be safe and are approved for use in humans, others such as phenformin and IACS10759 have shown high toxicity in clinical trials and hence are no longer under consideration as therapeutic agents [11, 14–16]. Our approach allows clustering of the potential OXPHOS inhibitors based on their chemotypes, providing an essential resource for identification of chemical features that are essential to consider when developing novel OXPHOS inhibitors that are both effective as well as safe to use in clinical settings.

## Results

### Data mining workflow for identification of novel OXPHOS inhibitors

**Fig. 1:**
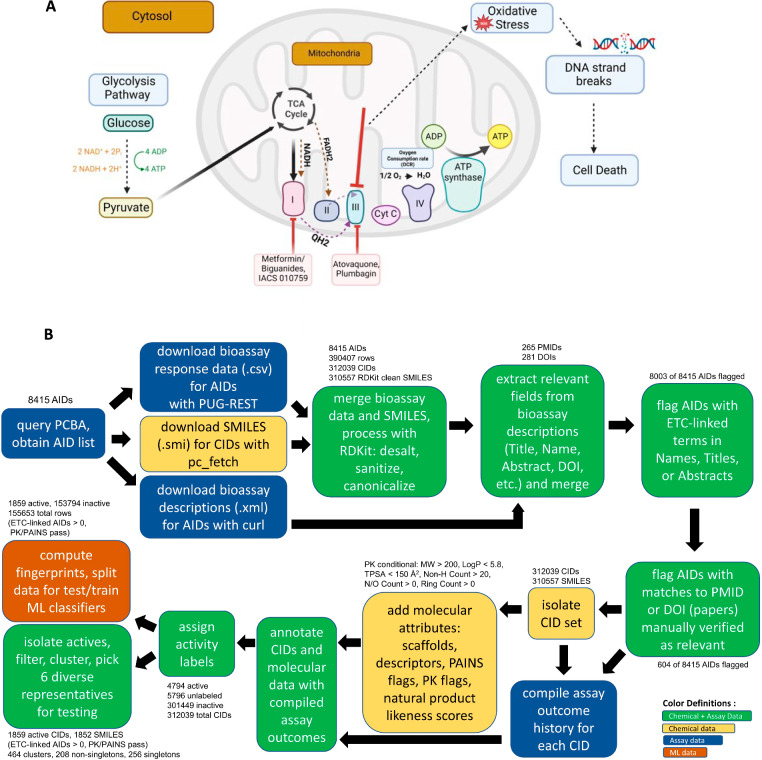
Data mining pipeline for identification of novel OXPHOS inhibitors. **A** The four multi-protein complexes (Complexes I–IV) of the OXPHOS pathway transfer of high energy electrons from NADH and FADH_2_ to oxygen. The proton gradient in the intermembrane space during this electron transfer drives the synthesis of ATP by ATP synthase. Pharmacologic inhibitors of Complex I and III block electron transport resulting in oxidative damage of DNA and other biomolecules causing cell death. **B** Data mining pipeline for the identification of OXPHOS inhibitors is shown. A list of 8415 OXPHOS-related assay ID (AID) was identified through a search of the PubChem database. The pipeline was then used to filter out 1859 candidates as potential OXPHOS inhibitors from a total of 310,557 compounds

In previous studies we identified several small molecule inhibitors of OXPHOS (Fig. 1A) that produce an oxidative stress condition that shrinks tumors without significant toxic effects in non-cancer cells [11, 12, 17–21]. These earlier efforts focused on a small set of α/β-unsaturated carbonyl-containing compounds that likely competitively inhibit ubiquinone interactions with ETC complexes or potentially engage in redox reactions to prevent effective transfer of electrons through the OXPHOS pathway in the mitochondria. To identify additional compounds and compound classes (chemotypes) that promote oxidative stress in cancer cells by inhibition of OXPHOS (Fig. 1A), we developed a novel data mining workflow (Fig. 1B).

First, a broad search was performed to identify potentially relevant AIDs from the PubChem database. The query returned 8,415 unique AIDs with matches to “electron transport chain,” “mitochondrial complex,” “mitochondrial respiratory chain,” or “mitochondrial membrane potential,” in their AID assay descriptions (Fig. 1B). The search was further limited to assays involving small molecule screens. Terms such as the tricarboxylic acid pathway were excluded to focus on assays directly associated with the electron transport chain (ETC) components of the OXPHOS pathway. The 8415 matching bioassay records contained a total of 390,407 individual molecule testing results involving 312,039 unique Compound record identifiers (CIDs). The PubChem SMILES for the 312,039 CIDs were downloaded and processed using RDKit to canonicalize, sanitize and strip counterions. These procedures reduced the set to 310,557 unique molecule structures (SMILES) with unique CIDs. Note that most compound testing records were concentrated in a small fraction of the total bioassay records. Only two AIDs contained more than 100,000 unique CIDs (AIDs 1465 and 540229). Just five AIDs had greater than 10,000 CIDs and 10 AIDs had greater than 100.

Given the large number of assay records returned in this initial search, a more stringent filter was applied to reduce the bioassay set to those most relevant to the OXPHOS pathway. Based on inspection of the assay titles, we developed a list of terms for a secondary filter consisting of 39 positive terms (involving electron transport in mitochondria) and 2 negative terms (related to the photosynthesis pathway) (Additional file 1). These terms were searched in the text of the assay title, description, and abstract of each AID. We anticipated that matches to these specific terms (or non-matches to the negative terms) would remove assays less directly associated with ETC mechanisms. These priority AIDs are referred to as “ETC-linked.” This secondary filter reduced the set to 8003 ETC-linked AIDs, involving 228,240 unique CIDs and 227,554 unique SMILES (Fig. 1B).

Given our goal of identifying drug-like OXPHOS inhibitory molecules as starting points for clinical development, the remaining compounds with non-drug-like characteristics were flagged. Both a Lipinski-like pharmacokinetics (PK) filter (MW > 200 g/mol, MolLogP < 5.8, total polar surface area < 150, number of heavy (non-hydrogen) atoms > 20, number of nitrogen or oxygen atoms > 0, and at least one ring) and PAINS (Pan Assay Interference Compounds) filter were applied.

The molecules were then labeled as OXPHOS-active or -inactive based on the PubChem activity outcome categories [22] within the 8003 ETC-linked bioassay records. Compounds were labeled as ‘active’ if they tested positive (PubChem Activity Outcome = = ‘Active’) in at least one assay and the number of positive assay results was equal to or greater than the number of negative testing results. Inactive compounds were defined as those that tested negative in at least one assay (PubChem Activity Outcome = = ‘Inactive’) and having no ‘active’, ‘inconclusive’, or ‘unspecified’ outcomes. All remaining compounds were designated “unlabeled” and considered inconclusive.

Based on our activity labeling, among the 227,554 unique molecules (228,240 CIDs) tested in the 8003 ETC-linked AIDs, there were 4140 active molecules (4199 CIDs). After applying PK and PAINS flags, the number of active molecules was reduced to 1852 (1859 CIDs). This “drug-like” active set was clustered based on structural similarity, yielding 464 unique chemical clusters (208 non-singletons). Structures of the medoids (most central compound in a chemical cluster) are shown in Additional file 2 to demonstrate their structural diversity. Additionally, the published records associated with these active compounds were identified. These records corresponded to articles with 74 unique PMIDs associated with active individual compounds from 297 AIDs. This result indicated that the algorithm used in our analyses was successful in reducing the set of relevant compounds (464 cluster representatives) by 670-fold from the original 390,407 compound testing records (312,039 CIDs and 310,557 unique compound structures) in the initial 8415 AIDs identified in the PubChem query.

### Data mining

**Fig. 2:**
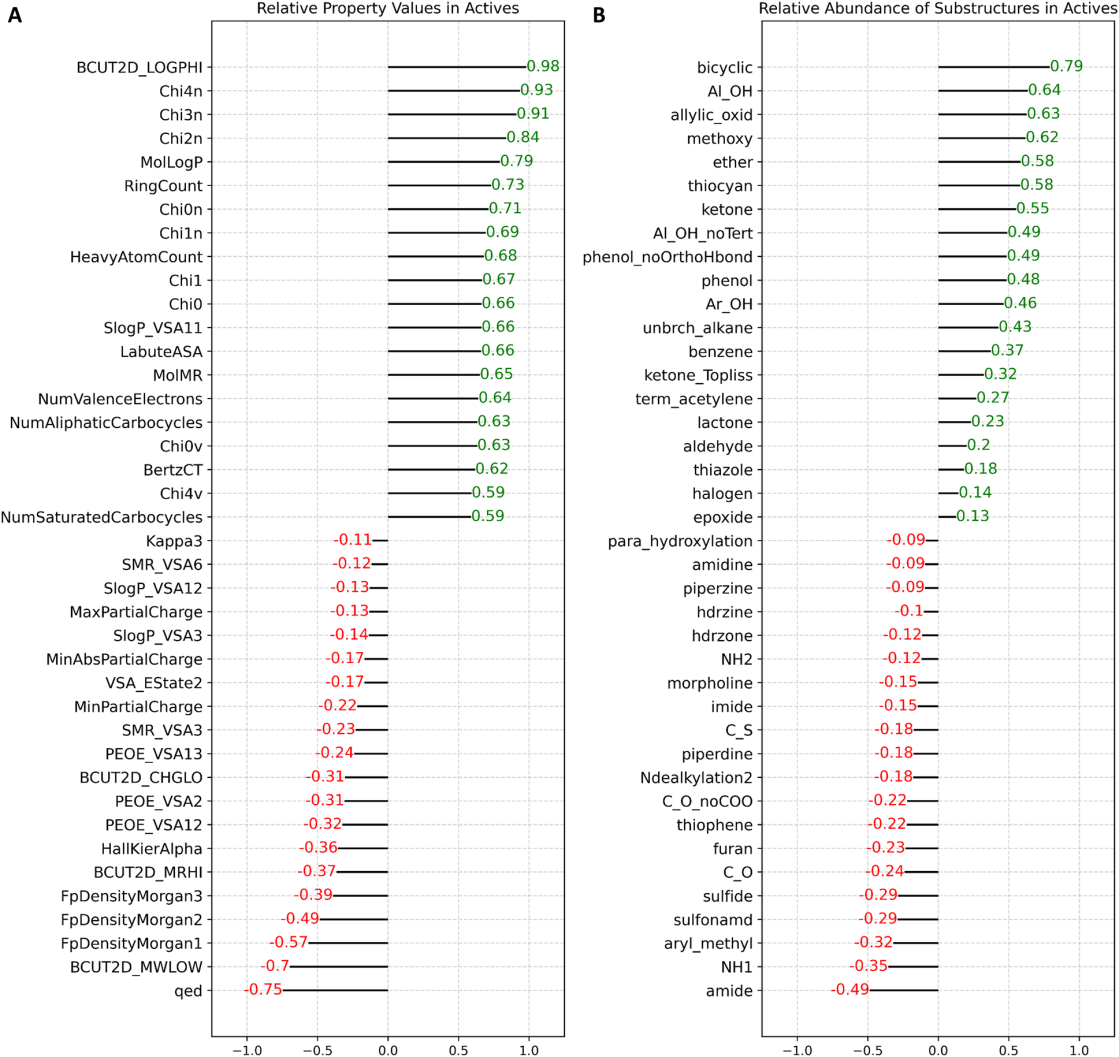
Active compounds in the OXPHOS assay data set diverge from inactive compounds with respect to **A** chemical properties and **B** specific chemical substructures/functional groups. In **A** and **B**, the diverging bar plot shows the difference between active and inactive compound population means after transformation by standard scaling (mean = 0, standard deviation = 1). Difference bars are shown for only the top 20 and bottom 20 most skewed molecular attributes with respect to OXPHOS activity. Green values indicate shifts in favor of OXPHOS actives. Red values show shifts favoring OXPHOS inactives. Definitions of fragments and properties are provided in Additional file 8

To identify distinguishing characteristics of OXPHOS inhibitor candidates, we compared both property and fragment/functional group distributions between the active and inactive compound populations (Fig. 2A and B). A subset of 155,653 unambiguously labeled molecules (153,794 inactive and 1859 active) derived from ETC-linked AIDs and that passed PK and PAINS filters were isolated for analyses. This set is referred to as the PC_OXPHOS training set. In terms of molecular properties, OXPHOS-active compounds exhibit elevated values for hydrophobicity-based features including BCUT2D_LOGPHI (highest eigenvalue for an atom adjacency matrix of elements as atom-wise logP contributions), MolLogP (predicted LogP), SlogP_VSA11, and MolMR (predicted molar refractivity). Active molecules also show relatively high values for size-related features like LabuteASA (van der Waals approximate surface area), number of atoms, molecular weight, number of rings, and aliphatic and aromatic carbocycles. OXPHOS actives also exhibit markedly lower Morgan fingerprint densities (molecular complexity) and quantitative estimate of drug-likeness (QED) values. Regarding structural characteristics, active compounds show higher abundance of bicyclics and oxygen-containing functional groups including ketones, allylic oxides (alpha/beta unsaturated carbonyls), hydroxyl groups (aliphatic, aromatic, and phenolic), and ethers. In contrast, amide and primary amine (NH1) functional groups show a notably lower than random prevalence.

### UMAP analysis to identify chemotype clusters of OXPHOS inhibitor candidates

**Fig. 3:**
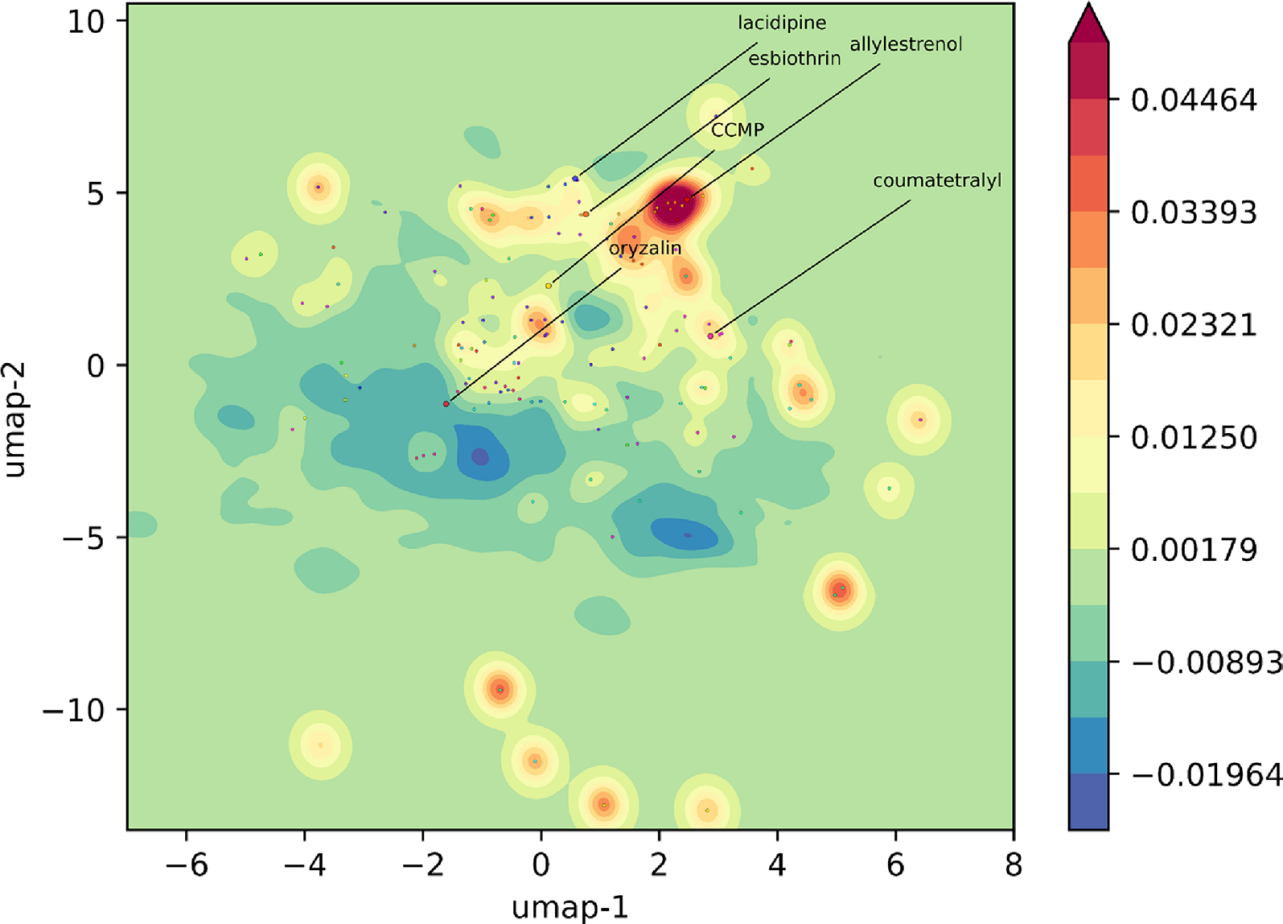
UMAP showing density differences between active and inactive compound distributions in a 2-D chemical space of PC_OXPHOS—an unambiguously labeled subset of compounds tested in OXPHOS assays (PC_OXPHOS). The difference map was derived by subtracting the KDEs of the active and inactive points. Positive values (red) show regions of relatively high OXPHOS-active density. Negative values (blue) show relatively low density for OXPHOS actives. Medoids from active chemical clusters of size 3 or greater are overlayed as labeled points. The 6 compounds we acquired and tested on OXPHOS-related endpoints are also shown as points labeled by compound names

To assess the diversity of the OXPHOS active chemotypes and the extent to which they might be structurally distinguished from inactive compounds in a low dimensional chemical space, we performed a 2D Uniform Manifold Approximation and Projection (UMAP) on the PC_OXPHOS training set using Morgan fingerprint representations. Using the compounds’ UMAP coordinates as data points, we plotted a contour map of the kernel density estimate (KDE) of the chemical space and then overlayed OXPHOS-active compounds as points on this space (Additional file 3A). Clusters larger than 2 compounds were colored with cluster medoids labeled by cluster ID numbers. All other active compounds (from doublets or singletons) were shown as unlabeled grey points. Contour maps of the KDE for OXPHOS-inactive (Additional file 3B) and OXPHOS-active (Additional file 3C) show substantial divergence in their locations within the chemical space. As might be expected with the marked class imbalance (1.2% active) in the PC_OXPHOS training set, inactive map looks nearly indistinguishable from the full compound distribution (Additional file 3A and B). However, the OXPHOS-active KDE shows many distinctive superclusters, groupings of related clusters, that depart from high-density regions of the general chemical space. The differences are more clearly illustrated in a difference map created by subtracting the inactive KDE from the active KDE (Fig. 3). The UMAP projection, a dimensionality reduction technique known to preserve local distance relationships, effectively co-localized cluster cohorts and mapped structurally related clusters into adjacent regions where fundamental chemotypes are shared within superclusters. For example, the highest density OXPHOS-active supercluster, consisting of clusters 1, 27, 28, 31, 37, 45, 46, 58, 65, and 66 (upper center-right of Fig. 3), mostly share a common steroid-like chemotype (a 4-ring poly-alicyclic scaffold) (Additional file 3D). Based on inspection of the UMAP projection, we categorized the OXPHOS-active densities into 3 broad chemical classes: (i) oxygen-rich, lightly unsaturated polycycles—which include the alpha/beta unsaturated groups, ketones, ethers, and alcohols (Additional file 3D), (ii) nitrogen-rich polycycles, including biguanide-like chemotypes with poly-nitrogenated rings, imine, and urea groups (Additional file 3E), and (iii) miscellaneous group that covers the remaining diverse structures (Additional file 3F). The oxygen-rich and nitrogen-rich groups share moieties with established OXPHOS-inhibitory agents like rotenone and metformin, respectively.

### Testing candidate molecules selected from data mining for OXPHOS inhibitory activity

Next, we conducted targeted assays to verify the OXPHOS inhibitory activity of randomly selected candidate compounds identified through our data mining approach. Six compounds, oryzalin, allylestrenol, esbiothrin, lacidipine, coumatetralyl, and (Z)-2-cyano-*N*-cyclohexyl-3-(5-morpholin-4-ylfuran-2-yl) prop-2-enamide (CCMP), each representing a unique chemotype from a distinct location in active UMAP space (Fig. 3), were chosen to verify their OXPHOS inhibitory activities. All six compounds were purchased from established vendors as high purity (> 95%) formulations in DMSO.

**Fig. 4:**
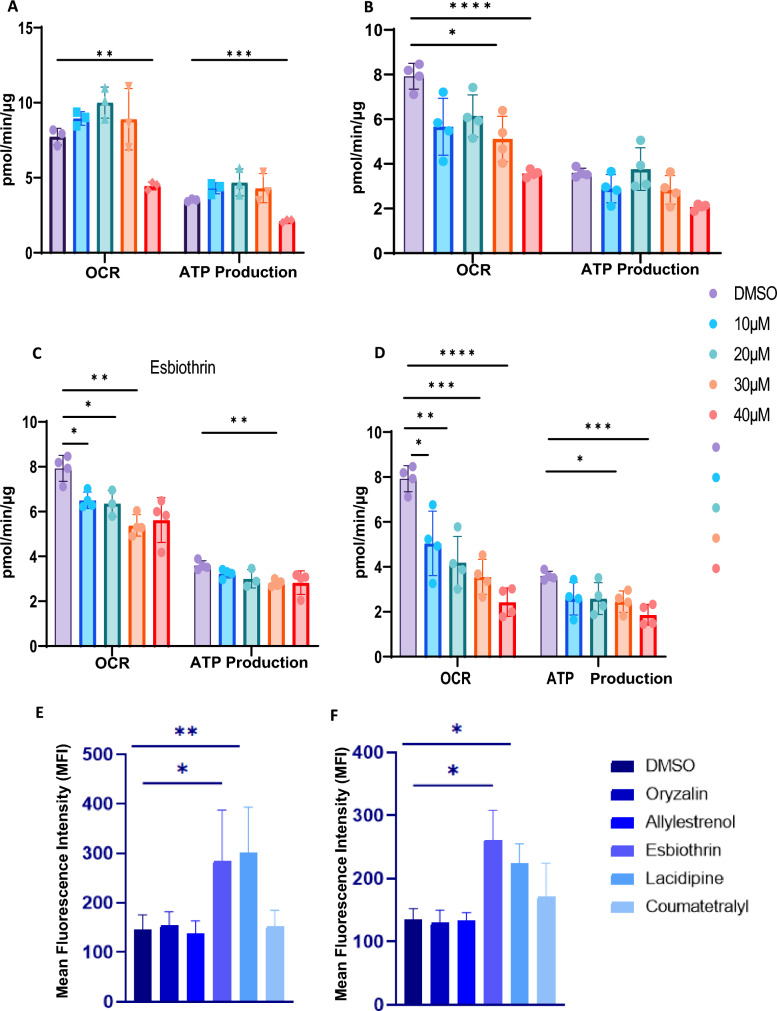
Oryzalin, allylestrenol, esbiothrin, and lacidipine inhibit OXPHOS and induce oxygen radical flux. **A**–**D**, ID8 cells preincubated with oryzalin, allylestrenol, esbiothrin and lacidipine (10–40 μM), respectively were examined in XFe96 Seahorse instrument for determination of oxygen consumption rate (OCR) and ATP production. DMSO was used as vehicle control in all experiments. Results shown above are the average of four technical replicates conducted with ID8 cell lines with *p* < 0.05 (**A**–**D**). **E**, **F**, ID8 and OVCAR-5 cells, respectively, were pre-labeled with the oxygen radical-sensing dye H2DCFDA were treated with oryzalin, allylestrenol, esbiothrin, lacidipine and coumatetralyl (10 μM) for 45 min. The increase in intracellular oxygen radicals was determined by flow cytometry (**E**, **F**). The bar graphs shown are representative of three biological replicates. The bar graphs show averages of the median fluorescence levels from the three technical replicates for each cell line. *p* < 0.05

OXPHOS inhibitory activity of the six test compounds was tested in bioenergetics assays conducted with the murine ovarian cancer cell line ID8 using the Seahorse XFe96 bioanalyzer. Cells were cultured in media with or without the drugs (0–40 μM) for 45 min prior to determining oxygen consumption rate (OCR), a measure of OXPHOS activity. Four of the six compounds tested, oryzalin, allylestrenol, esbiothrin, and lacidipine inhibited OCR in the ID8 cells (Fig. 4A–D). Coumatetralyl and CCMP did not inhibit OCR production in these assays indicating that they were not functioning as OXPHOS inhibitors (data not shown). In these experiments, lacidipine mediated the strongest dose-dependent inhibition of OCR in the ID8 cells (Fig. 4D). This decrease in OCR was complemented by inhibition of ATP production in the ID8 cells treated with oryzalin, allylestranol, esbiothrin, and lacidipine (Fig. 4A–D). Here again, among the six compounds tested, lacidipine was the strongest inhibitor of ATP production in ID8 cells (Fig. 4D).

In previous studies, we demonstrated that known OXPHOS inhibitors, atovaquone, plumbagin and others, by interfering with electron transport, induce a rapid intracellular oxygen radical flux [11, 12, 17, 18, 23]. This increase in the reactive oxygen radicals causes severe cellular damage leading to cell death. With four of the six test compounds demonstrating OXPHOS inhibitory activity, we next tested their ability to induce an intracellular oxygen radical surge using an established flow cytometry-based assay. The human and murine ovarian cancer cell lines, OVCAR-5 and ID8, respectively, were prelabeled with the oxygen radical sensing dye, H2DCFDA. Upon oxidation by reactive oxygen species, H2DCFDA is converted into green fluorescent DCF that is detected by flow cytometry. Using this assay, we noted that esbiothrin and lacidipine induced reactive oxygen flux in both OVCAR-5 and ID8 cells (Fig. 4E and F). While CCMP could not be tested in this assay because of its inherent fluorescence that interfered with that of H2DCFDA, oryzalin, allylestrenol, and coumatetralyl did not induce an increase in intracellular oxygen radicals.

**Fig. 5:**
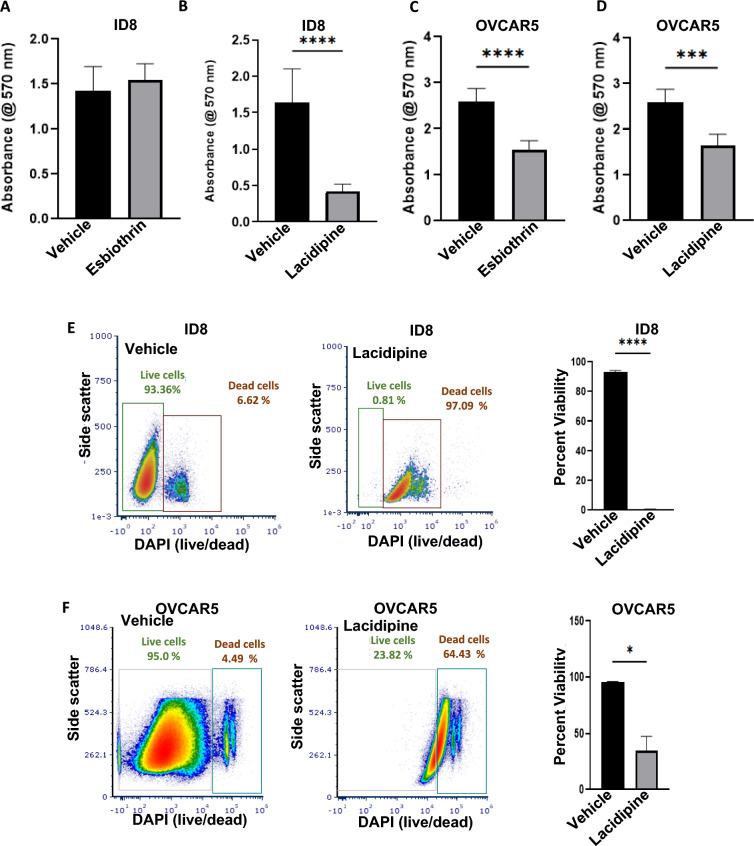
Lacidipine and esbiothrin cause cancer cell death. A-D, ID8 (**A** and **B**) and OVCAR-5 (**C** and **D**) cancer cell lines were treated with esbiothrin (**A** and **C**) and lacidipine (**B** and **D**) at 10 μM (gray bars) or with vehicle (DMSO, black bars) for 72 h and proliferation of the cell lines was determined using MTT assay. The assays were conducted in triplicate and in each experiment, there were eight technical replicates of each condition tested. The plots show average data from the three biological replicates for each cell line. *p* < 0.05. E and F, ID8 (**E**) OVCAR5 (**F**) cells were treated with lacidipine (10 μM, gray bars) for 72 h and stained with DAPI to assess cell death by flow cytometry. Aggregate data from three technical replicates are shown in the bar graphs for each cell line

Finally, we tested the chemotoxic effects of the six candidate compounds against OVCAR-5 and ID8 cells. In these assays, oryzalin, allylestrenol, coumatetralyl, and CCMP did not mediate statistically significant decrease in the viability of OVCAR-5 and ID8 cells after 72 h treatment (data not shown). Treatment with esbiothrin had no effect on viability of ID8 but resulted in significant decrease in viability of OVCAR-5 cells (Fig. 5A and C). On the other hand, lacidipine showed consistent and statistically significant reduction in viability of both OVCAR-5 and ID8 cells after 72 h of treatment (Fig. 5B and D).

Since consistent decrease in viability was observed with lacidipine treatment, we further tested this drug for its ability to induce cell death using a flow cytometry-based assay. In this assay, OVCAR-5 and ID8 cells were treated with lacidipine. After 72 h of incubation, the cells were labeled with DAPI, a dye that percolates through pores that form in cell membranes of dying cells. We observed that the majority of the ID8 (~ 97%) and OVCAR-5 (~ 64%) died following exposure to lacidipine (Fig. 5E and F). These experiments identified two (lacidipine and esbiothrin) of the six randomly selected compounds as potential candidates for further testing against ovarian cancer.

### Pipeline-aggregated bioassay data train effective machine learning classifiers of OXPHOS inhibitors

Besides, enabling selection of candidate compounds from historical public assay data, our data mining pipeline provides rich data for training machine learning classifiers to prioritize novel compounds for testing. We observed that classifiers trained with these data perform well on predicting OXPHOS inhibitors in retrospective testing based on standard virtual screening metrics. Three standard machine learning classification models, commonly used for ligand-based virtual screening tasks, were developed using the PC_OXPHOS data subset compiled by the pipeline: logistic regression (LR), random forest (RF), and support vector machine (SVC) classifiers. For each model type, standard chemical fingerprints and/or RDKit molecular descriptors were used as compound input representations. Model hyperparameters were explored in fivefold cross-validation on 85% of the data. The top performing model from each combination of classifier type and input representation (Additional file 4) was then evaluated on a held-out test set comprising 15% of the set (Table 1). The RF and SVC models perform very well in both classification metrics F1 and MCC as well as in standard virtual screening metrics ROCAUC, average precision (like PRAUC), and EF1, which depend only on the rank order based on model scoring.

**Table 1:**
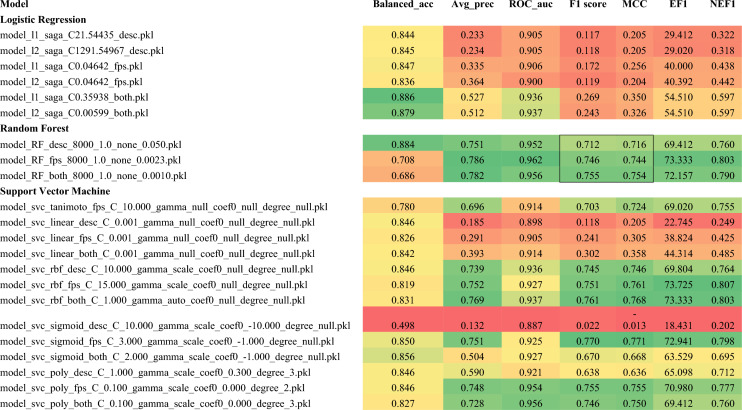
Performance of machine learning models used to predict OXPHOS inhibitors

For another retrospective test of our best models, we identified known OXPHOS inhibitors from literature outside of our training set and examined our performance in virtual screening to retrieve these compounds within random sets of inactive compounds from our test set. We compiled 51 documented OXPHOS inhibitors (Additional file 5) from which 7 were removed due to overlap with the training set (boldface). Given the remaining 44 novel actives and a target baseline active fraction of 1.2% as observed in the training set, we shuffled these documented actives in with subsets of 3658 inactives randomly drawn from the test set. Both fingerprint-based RFC and SVC were evaluated on 10 sets composed with different inactive sampling (Table 2). Both models fail to retrieve any of the known positives based purely on classification label. However, most of the known actives receive high scores from the models. Thus, the models show excellent potential in virtual screening applications that rely on score-based ranking rather than discrete prediction labels. For the RFC, the ROCAUC and average precision were 0.900 (0.002) and 0.303 (0.019), respectively. The EF1 was 29.4 (4.4). As a comparison, from a random selection of 1% of compounds (37) from a set of 3702 compounds, one should expect on average 0.44 actives (fewer than 1); with hit enrichment provided by the RFC prioritization, one should expect 11–15 active compounds among the top scoring 37 (30–40%). The SVC performed worse with respect to ROCAUC and average precision, but slightly better with respect to the EF1 metric. Both RF and SVC classifiers perform worse than observed on the test set (Table 1).

**Table 2:** Virtual screening model performance on compound sets containing known OXPHOS inhibitors

Metric	RFC-fps		SVC-fps (RBC)	
	Mean	stdev	Mean	stdev
ROCAUC	0.900	0.002	0.823	0.003
Avg Prec	0.303	0.019	0.275	0.019
EF1	29.4	4.4	32.0	1.3
NEF1	0.405	0.060	0.441	0.017

Classifiers like LR, RF, and SVC are readily interpretable and thus facilitate further elucidation of the key molecular features, those most informative in discriminating OXPHOS active and inactive compounds. Feature importance analyses on our top-performing descriptor-based and fingerprint-based RF models were carried out using two methods: Mean Decrease in Impurity (MDI) and feature permutation (Tables 3 and 4). MDI (or Gini Importance) scores features based on the frequency with which a given feature appears at nodes among all of the RF trees, weighted by the number of samples split by those nodes [24]. MDI analysis of the descriptor- and fingerprint-based RF models reveals key features, which appear highly correlated with the features’ absolute mean divergences between active and inactive populations (Fig. 2A and B) with respect to both descriptors (0.450) and fingerprint bits (0.908) (Additional file 6). Feature importances were also evaluated by feature permutation, a model-agnostic approach that reflects the decrease in model performance (here average precision) when a given feature’s values are randomly shuffled on a test set and then its samples re-evaluated. When ranking the features by permutation importance, we observed lower correlation with the absolute feature divergence values (0.005 sand 0.358 for descriptors and fingerprints, respectively) (Additional file 6). We take permutation as the more reliable assessment since the approach evaluates on data outside of the training set and is known to be less likely to inflate high cardinality features like MDI [25]. Nevertheless, MDI and permutation methods were correlated (0.562 and 0.351 for descriptors and fingerprints, respectively) with substantial agreement among their highest ranked features based on percentile ranks in Tables 3 and 4.

**Table 3:**
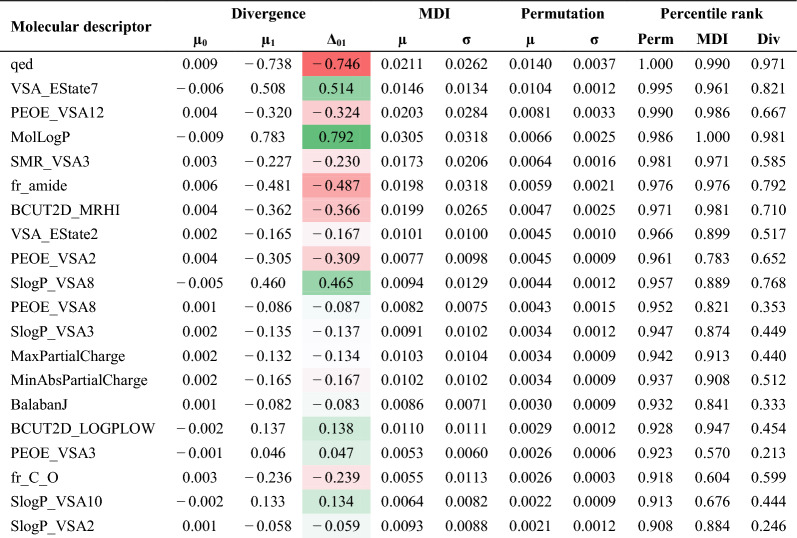
Feature Importances for Molecular Descriptors

**Table 4:**
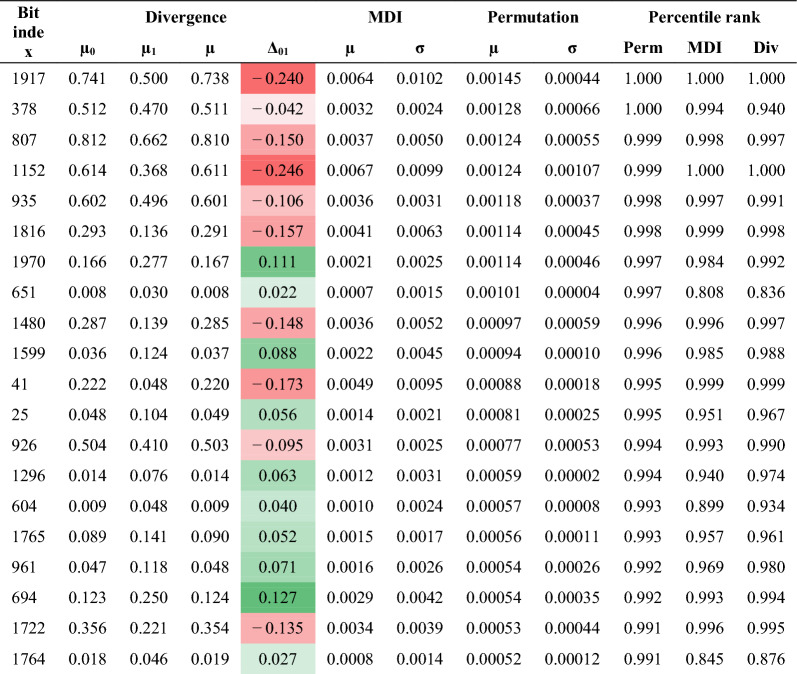
Feature Importances for ECFP6 Fingerprint Bit Indices

For descriptor features, quantitative estimate of drug-likeness (QED) and MolLogP are among the most important. In RF and other ensemble models, information provided by a feature is dependent on its relationship to other features and so a direct positive or negative influence on the classification score is not revealed by the importance scores in the methods used and likely oversimplifies a feature’s influence in the model. However, based on the direction of shift among the strongly divergent features, it appears that QED has a profoundly negative impact on OXPHOS activity, whereas elevated MolLogP has a positive impact. Other highly ranked property-based descriptor features include several composite features involving parameters like sLogP and PEOE (partial charge contributions) with respect to van der Waals surface area (VSA) contributions—see https://greglandrum.github.io/rdkit-blog/posts/2023-04-17-what-are-the-vsa-descriptors.html for discussion on their derivations. Notably, BCUT2D_MRHI, a complex descriptor based on the largest eigenvalue for an atom adjacency matrix of empirical atom-wise molar refractivity contributions has a strong negative shift in the divergence. Interpretation of these complex descriptors is not straightforward. The extent to which a parameter like BCUT2D_MRHI relates to physical molar refractivity, which in turns relates to polarizability and molecular weight, is not clear.

**Fig. 6:**
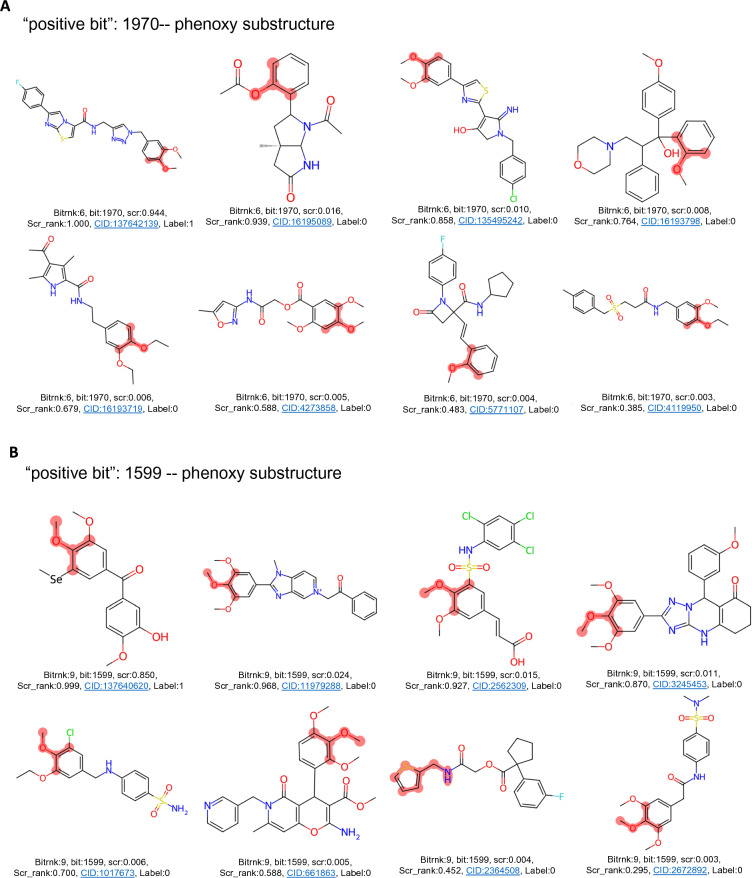
Important ECFP6 bits **A** 1970 and **B** 1599, corresponding to phenoxy-type substituents, are highlighted on 8 compounds drawn from the PC_OXPHOS set. The 8 example compounds representing the given bit were intentionally selected to span the range of scoring by our RFC model. Bits 1970 and 1599 were particularly abundant in OXPHOS-active compounds. The red highlighted atoms and bonds correspond to the substructures represented by each bit. Under each compound several parameters are reported. *Bitrnk* is the ranked importance of the bit according to permutation analysis. *Bit* is the bit index within the ECFP6 fingerprint (length = 2048). *Scr* is the score observed for the compound by our top performing RFC. *Scr_rank* is the percentile ranking of the score for the compound. *CID* is the PubChem Compound Identifier. *Label* is the ground truth label for the compound (1 = active, 0 = inactive)

Fragment-based descriptors might be more directly interpretable. Amides and generic carbonyl groups (including within carboxylate groups) are strong, likely negative, features for OXPHOS inhibition activity (ranking 6th and 18th overall based on permutation analysis). However, more specific carbonyl types represent the strongest positive fragment descriptors: allylic oxide (fr_allylic_oxid) and ketones (ranking 40th and 50th). Important ECFP6 fingerprint bits/substructures, when mapped visually onto compound structures, are congruent with our calculated descriptor fragment importances. Based on the permutation importance rank order of bits in ECFP6 fingerprints, phenoxy substructures were mapped to positive bits 1970 and 1599 (Fig. 6A and B). Another high-importance positive bit, 694, maps to a general methine carbon (e.g., alkenes, carbonyl, and imine bonds) (Additional file 7A). Prominent negative bits, 1917 and 1152 map to carbonyl and secondary amine substructures (Additional file 7B and C), respectively. Note, some collisions are observed at certain bits, reflected by different substructures mapping to the same bit index. However, we observe this to be infrequent and should not severely complicate interpretation of bit importance, given our fingerprint length (2048) and resulting bit density.

## Discussion

Here, we report the successful development of a pipeline for data mining of public assay records in PubChem to identify diverse small molecule chemotypes with high OXPHOS inhibitory potential. The data compiled by the pipeline were also useful for training LBVS models for predicting OXPHOS inhibition. Our in-silico approach requires practically no new resource investment in assays to cull a total of 312,039 CIDs to a final hit list of 1859 CIDS (1852 unique parent SMILES) that not only have high potential for activity as OXPHOS inhibitors but also pass PAINS filters and possess generally good PK properties for oral bioavailability. Biological testing of six compounds selected from distinct active chemotypes shows a high hit rate as (a) four of these compounds inhibited OXPHOS (via measurements on OCR and ATP levels) and (b) two of the four compounds that inhibited OXPHOS also elevated intracellular oxygen radicals and decreased the viability of ovarian cancer cell lines. With the majority of the 1859 compounds identified through our datamining pipeline still to be tested, it is likely that several additional OXPHOS inhibitory candidates can be identified as potential therapeutic candidates for ovarian and other cancers.

Furthermore, the pipeline revealed distinctive chemical features of OXPHOS inhibitors, provided a breakdown of diverse OXPHOS-active chemotypes (464 clusters) observed across 8415 public assay records (Additional file 2), mapped these to specific OXPHOS-active high-density regions within the broader chemical testing space, and provided a rich data for training classifiers that performed well in retrospective tests and should have future applicability in prioritizing OXPHOS inhibitor candidates within new compound sets.

### Observations on OXPHOS inhibitory chemotypes and their chemical characteristics

Our data mining pipeline retrieved a substantial fraction of compounds from a set of documented OXPHOS inhibitors we compiled from literature. Among the 51 inhibitors (Additional file 5), 20 of these were in the full compound set (312,039 unique CIDs) based on canonical SMILES matches. Of these, 10 were appropriately recognized as active based on our pipeline conditions and assigned positive labels: atovaquone, plumbagin, FCCP, alpha-TOS, antimycin A, rotenone, curcumin, deguelin, Atpenin A5, and fenofibrate. However, our activity labeling conditions also resulted in 3 documented inhibitors being mislabeled as “inactive:” phenformin, citral, and 3-nitropropionic acid. Furthermore, several known actives had mixed results in the compiled assay records and were considered “unlabeled.” These included metformin, diphenylamine HCl, lonidamine, amobarbital, tiabendazole, papaverine, and ranolazine. For purposes of compound prioritization and training machine learning models, we favored stringent labeling assignment conditions so that positive and negative instances were unambiguous with respect to assay outcomes in their associated AIDs. However, the cause of these apparent mislabels is not clear, potentially resulting from assay sensitivity or error, inclusion of non-relevant assays, excessive stringency of labeling criteria, or other issues. Nevertheless, given the high false positive/negative rates in primary screening data, and the large set of disparate assays from which we collected data, we are encouraged that 10 of the 20 documented inhibitors in the mined data set were assigned active labels and another 7 were considered inconclusive. Note that active labeled compounds represented only 1.5% of the initial data set (4794 actives in 312,039 total CIDs).

Among the 20 documented inhibitors identified in the original data set, all had at least one ETC-relevant assay record. Interestingly, 11 of these 20 fail the PK filters and 3 fail the PAINS filter: atovaquone (adjacent carbonyls), plumbagin (quinone), and FCCP (imine and cyano groups). Of the 20, only 10 were assigned active labels based on activities across compiled assay records, and just 5 were ultimately included in the final 1859 actives after passing PAINS, PK, and ETC-relevance conditions: rotenone, curcumin, deguelin, atpenin A5, and fenofibrate. Rotenone and deguelin are multicyclic structures with α, β-unsaturated carbonyls. Curcumin, and Atpenin A5 are monocycles with a α,β-unsaturated carbonyls. Fenofibrate has a ketone group bridging 2 different phenyl substituents. Rotenone, deguelin, and fenofibrate target Complex I in ETC and have documented anti-proliferative effects whereas Atpenin A5 targets Complex II and exhibits cardioprotective effects (reviewed in [13, 26–29]).

Compounds with mitochondrial bioavailability and OXPHOS activity tend to possess distinct physicochemical features. A previous study involving models for predicting mitochondrial toxicity showed significant enrichment for mitochondrial toxins in the high SlogP range of 4–9 [30]. Toxins also had a higher abundance of aromatic rings/aromatic carbocycles, higher molecular weight and surface areas (LabuteASA), and lower fraction sp^3^ hybridized carbons. Our analysis confirms most of these earlier findings, further supporting unique property ranges for compounds intended for distribution to the inner membrane of mitochondria. In terms of RDKit physicochemical descriptors, OXPHOS actives exhibit relatively high MolLogP, RingCount, Labute ASA, MW, and NumAromaticCarbocycles, (Fig. 2A). NumAromaticCarbocycles and MW are not shown but were shifted right by 0.357 and 0.580 standard deviations, respectively. Contrary to a previous report where mitotoxins were shown to possess a lower fraction of Csp3 carbons, we observed a very small increase in the fraction Csp3 (0.059) in OXPHOS actives [27]. Fragment analysis revealed enrichment for bicyclic, phenol, Ar-OH, and benzene substructures (Fig. 2B). Whereas previous 2-D PCA projections did not show notable separation of mitotoxins from non-toxins [30], our analysis via 2-D UMAP on a much larger set of compounds did show clear distinctions in OXPHOS-active and -inactive distributions within chemical space (Fig. 3).

### Observations on testing of 6 prospective OXPHOS inhibitors

The six compounds that we tested represent distinct chemotypes. While four of these six compounds showed OXPHOS inhibitory activity, it is important to note that one of the compounds from this group, esbiothrin, is a known pesticide and hence is likely to present an unacceptable toxicity profile in human patients. This situation can be conveniently remedied in future iterations of our data mining algorithm by including additional filters that specifically exclude compounds that are known to be pesticides, insecticides, or those with a reported high toxicity in humans or in experimental animals.

Among the six compounds we have tested, lacidipine is especially promising as it exhibits higher activity as an OXPHOS inhibitor and shows clear chemotoxic activity against cancer cells. Extensive studies have demonstrated lacidipine as an antagonist of the L-type calcium ion channels and because of this function, lacidipine is used for the treatment of hypertension. Our data mining approach and the data from the biological assays demonstrate for the first time a novel biological activity for lacidipine.

Our data mining pipeline reveals that OXPHOS inhibitory chemotypes are diverse (represented by 464 total structural clusters (Additional file 3A) and 135 larger clusters (greater than 2 members) (Additional file 3B). However, our UMAP projection (Fig. 3) shows that these clusters tend to localize to specific, confined regions of the chemical testing space in superclusters. Inspection of the compound structures within these hot spots confirms the chemotypes are highly related within these regions. This mapping and confinement of the OXPHOS-active space might be leveraged for focusing screening efforts for other OXPHOS inhibitors. Such mappings may also serve as a basis to more deeply characterize the OXPHOS structure–activity relationship (SAR) of active chemotypes—perhaps determination of their specific biochemical targets within the OXPHOS pathway. Importantly, this analysis also identifies the chemical space that is productive when targeting OXPHOS pathway.

### Limitations in data mining prototype and planned advancements

Given our ongoing effort to develop drug candidates for treatment of ovarian cancer, we believe the pipeline might be improved by addressing some limitations exposed in prototyping here. In compound prioritization, greater emphasis should be placed on achieving PK/tox objectives, in addition to OXPHOS inhibitory potential, to facilitate faster translation to in vivo testing. Perhaps, priority should be given to compounds from OXPHOS-active chemotypes represented by already approved drugs or known metabolites. Also, many of the OXPHOS active agents collected in data mining were later observed to have known liabilities with respect to toxicity. This might be something gleaned directly from CID records, which are frequently annotated with toxicity data. Additionally, more stringency might be beneficial in AIDs by requiring cancer-related terms like anti-cancer, anti-proliferative, anti-tumor, cell killing, etc. Further, since OXPHOS, mitochondrial function, and oxidative stress are terms likely associated with toxicity- or counter-screens, an effort should be made to flag AIDs involving toxicity screens. For example, AIDs involving healthy tissues/normal cells (for e.g., fibroblasts or PBMCs), or in animal models and cell death read-outs based on dye reagents for assessing cell penetrance or DNA binding [31], should be flagged as such. Inclusion of an additional toxicity label may enable multi-objective prioritization based on both potential for OXPHOS activity and potential toxicity.

Another key issue we hope to address stems from the fact that the OXPHOS inhibitors identified in our data mining pipeline act at different points in the electron transport chain, or perhaps even outside of the chain. In fact, some of the agents have been characterized with respect to their targeted complexes within OXPHOS [13]. The desired oxidative stress condition or metabolic perturbation, and thus the anti-cancer effect ultimately promoted by the agents, likely depends on their specific biochemical targets in the chain. Therefore, we hope to advance this work by elucidating the SAR between chemotypes and points of intervention in the OXPHOS pathway. Such efforts are likely critical for improving prioritization of candidate compounds with the highest potential as starting points for chemotherapeutic agents for the treatment of ovarian and other cancers.

## Conclusions

We have successfully developed a data mining and machine learning-based approach for efficient identification of OXPHOS inhibitors. In a data set mined from PubChem, we observe a broad range of potentially OXPHOS inhibitory chemotypes, exhibiting signature chemical substructures and properties. Using UMAP with fingerprint representations, these active chemotypes concentrate in specific regions of chemical space. Testing of six compounds taken from distinct active regions in the OXPHOS-active UMAP space resulted in 2 compounds that promote oxidate stress conditions, one of which with anti-proliferative activity in ovarian cancer cell lines. Here we demonstrate that our data mining approach enables highly focused screening for OXPHOS inhibitors with resulting anti-cancer activities. This prototype pipeline is extensible and could be adapted for focus screening on other phenotypic targets for which sufficient public data are available.

## Methods

### Extraction and processing of PubChem bioassay data

The complete bioassay data extraction and mining workflow to identify OXPHOS inhibitor chemotypes is shown in Fig. 1B. The pipeline was implemented on an Ubuntu 20.04.6 LTS workstation through a sequence of linux bash shell and python scripts (Python 3.8.10). The conda environment (.yaml), scripts, and documentation are available on GitHub: https://github.com/SpencerEricksen/PubChem_DataMining_OXPHOS. The main modules (versions) used were RDKit (v2022.09.1), pandas (v1.5.3), and scikit-learn (v1.2.2). The pipeline was initiated by submitting the following query to PubChem via the browser-based advanced assay search (https://www.ncbi.nlm.nih.gov/pcassay/advanced) on Feb 22, 2022, 13:57 CST):

- “electron transport chain”[Assay Description] OR
- “mitochondrial complex”[Assay Description] OR
- “mitochondrial respiratory chain”[Assay Description] OR
- “mitochondrial membrane potential”[Assay Description]) AND
- small_molecule[filt]

The query returned a list of matching Assay IDentifier (AID) records having the OXPHOS-related search terms in their bioassay description text. The number of matching AIDs (8415) exceeded the number of full AID records permitted for download in a single request (1000) so the matching AID information was saved using the “Send to” option to a text file (“pcassay_result.txt”). This text file includes a list of AIDs with associated assay titles, data source, number of substance records (SIDs) tested, and the number of active SIDs in the assay. Using a list of the AIDs from this file, the complete assay records–including compound records tested (CIDs) with associated assay outcomes–were downloaded (.csv) using a python wrapper to initiate URL-based requests through PUG-REST for each AID in the list.

The AID data sets were then merged into a single dataframe (bioassay dataframe) where each row represents a single compound testing record within a specific assay. Then, an xml-style SMILES pc_fetch file (.cgi) was created for the union of CIDs in the dataframe and submitted via PUG REST to fetch the SMILES. Returned SMILES were then merged into the bioassay dataframe using the CID as they key. Using the RDKit python cheminformatics toolkit, the PubChem SMILES were canonicalized, sanitized, and stripped of smaller counter ions. Some counter ions were not included among standard salt counter ion definitions in RDKit and so a secondary salt stripper function was applied that retained only the largest molecular fragment in cases where multiple fragments remain present in a SMILES. The RDKit-canonicalized SMILES, both salt and desalted forms, were stored in the bioassay dataframe as additional columns.

Next, bioassay descriptions and metadata associated with each AID were downloaded from PubChem in.xml format using curl within a BASH wrapper. From each AID’s.xml file, the following bioassay fields were extracted and merged into the bioassay dataframe using the AID as the key: ‘id’, ‘pmid’ (PubMed Identifier), ‘DOI’ (Digital Object Identifier), ‘Year’, ‘ChEMBL Target Name’, ‘ChEMBL Target Type’, ‘Target ChEMBL ID’, ‘Confidence’, ‘Relationship Type’, ‘Title’, ‘name’, and ‘Abstract’.

To prioritize the most relevant assays, AIDs that specifically involved electron transport chain (ETC) protein targets or ETC-associated functions were flagged. ETC-linked AIDs were identified by searching text from each AID’s name, title, and abstract for matches to a list of 39 positive ETC-related terms and 2 negative, photosynthesis-related terms (Additional file 1). A list of PubMed IDs (PMIDs) associated with these ETC-linked AIDs was generated. The papers (PMIDs) were examined for experimental relevance to ETC. Where the papers were confirmed to be ETC-relevant, the AID was given highest priority and flagged as having an ETC-linked PMID. For purposes of data mining and classifier training, only data from ETC-linked AIDs were used but an ETC-linked PMID was not required.

The set of CIDs in the bioassay dataframe and their corresponding desalted, RDKit-canonicalized SMILES were then isolated for generation of structural properties and features. Using primarily RDKit methods, each compound was assigned its Bemis-Murcko and reduced Murcko scaffolds, natural product-likeness score [32], PAINS flags using RDKit substructure matching against list of PAINS substructures (https://github.com/iwatobipen/rdkit_pains [33, 34], RDKit molecular descriptors (Additional file 8A), Morgan (circular) chemical fingerprints of length 2048 and radius = 3 [35], and a conditional Lipinski-like PK property flag based on desirable physicochemical constraints typical in hit-to-lead workflows [36]: molecular weight > 200 Da, mLogP < 5.8, total polar surface area < 150 Å^2^, fewer than 20 heavy atoms (non-hydrogen), at least one nitrogen or oxygen atom, and at least 1 ring.

Next, activity outcomes and scores within the 8415 OXPHOS-related AIDs were compiled and associated for each unique CID. Based on the compiled activity data, an activity label was designated for each CID. CIDs with at least 1 ‘Active’ outcome and a number of active outcomes greater than or equal to the number of inactive outcomes were considered OXPHOS-active molecules (inhibitors). Molecules with at least one inactive outcome and no active, unspecified, or inconclusive outcomes were designated as OXPHOS-inactive. These labels were used for data mining and machine learning procedures.

### Clustering of OXPHOS-active compounds

To examine the chemotypes active specifically in OXPHOS inhibitory function, the set of compounds (unique PUBCHEM_CIDs) labeled as OXPHOS-active, derived from ETC-linked AIDs, and passing PAINS and our PK filters were clustered by structural similarity. Hierarchical agglomerative clustering was performed using SciPy’s (v1.5.3) average linkage implementation. A condensed distance matrix was obtained based on chemical fingerprints as compound representations. As input to clustering, a pairwise distance matrix was computed for these compounds using the Jaccard distance between fingerprints (2048-length bit vectors). Medoid molecules for each cluster were selected as the compound having the lowest sum of distances to all cluster cohortss. The distance cutoff used for cluster assignment was 0.750. That means clusters with centroids nearer than 0.750 in Jaccard distance were merged into the same cluster. Based on the clustering outcome, the cluster IDs and cluster medoid flags were merged with the existing compound data.

### Development and evaluation of machine learning classifiers

LR, RFC, and SVC models were developed with training data produced by the data mining pipeline. Starting with the complete set of unique compounds from the data mining pipeline (312,039 compounds), a subset of 155,653 unique compounds were isolated (PC_OXPHOS), having definitive positive (1859) or negative (153,794) activity labels (‘label’ = 0/1), satisfying PK and PAINS conditions, and associations with at least one ETC-linked AID.

Molecules in this set were featurized as RDKit ECFP6 (length 2048, radius = 3) fingerprints and RDKit molecular descriptors. The “Ipc” descriptor from RDKit was removed due to scaling issues. Standard scalers were fit to training data to transform raw feature values into z-scores (mean = 0, standard deviation = 1). The standard scalers were saved and applied later to features in test and calibration sets prior to input for model evaluation.

The data were then split with 85% (132,305 total, 1604/130701 active/inactive) reserved as a training set for model hyperparameter determinations and 15% diverted as a test set for subsequent classifier evaluation. Grid-based hyperparameter searches were performed for each model type as indicated in Additional file 4. Models were generated using fingerprint inputs, descriptor inputs, and both. All models used SciKit-Learn implementations save for a bespoke Tanimoto kernel (https://github.com/gmum/pykernels/blob/master/pykernels/regular.py) used in a fingerprint-based SVC model (no standardization). Best performing hyperparameter sets were selected based on the sum of ROCAUC and Average Precision summary metrics observed in fivefold CV on the training data. Models were evaluated on the test set using standard classification performance metrics, F1score (F1) and Matthew’s Correlation Coefficient (MCC), as well as common ranking-based, virtual screening metrics: average precision (AP), area under the Receiver-Operator Characteristic Curve (ROCAUC), enrichment factor 1% (EF1), and normalized enrichment factor 1% (NEF1) [37].

Due to the large class imbalance in the data set (1.2% active), the RFC models required calibration for proper assessment of threshold-dependent metrics like F1 and MCC. LRs and SVCs did not require calibration. To calibrate RFCs, the data were split 3 ways into a training (70%), calibration (15%), and test (15%) sets. RFCs were trained on the smaller training set (70% vs. 85%) and then calibrated using SciKit-Learn’s CalibratedClassifierCV class (‘prefit’ = True) on the calibration data set. Calibrated RFCs were then applied on the test set to evaluate F1 and MCC performance metrics.

### UMAP analysis

To visualize and compare active and inactive compound distributions in chemical space, ECFP6 fingerprints from the same molecule set used for ML testing and evaluation were projected into 2 dimensions using the umap-learn python implementation of Uniform Manifold Approximation and Project (UMAP) analysis [38]. A grid-based search for suitable *n_neighbors* and *minimum distance* parameters was performed over the following space: {*n_neighbor*s: 5, 10, 15, 20, 25, 30, 40, 50, 100, 200} and {*minimum_distanc*e: 0.001, 0.025, 0.05, 0.10, 0.15, 0.20, 0.25, 0.30, 0.40, 0.50}. Based on inspection of the resulting maps and co-localization of cluster cohorts, suitable parameters were found to be *n_neighbors* = 50 and *minimum_distance* = 0.25. For contour maps, kernel density estimates for each compound set (all, inactives, actives) were calculated using the gaussian_kde function in Scipy’s stats module.

### Cell lines and reagents

The human ovarian cancer cell line, OVCAR-5, was obtained from ATCC, along with ID-8 cells (mouse ovarian cancer cell lines). These established cell lines were maintained in their respective recommended media and maintained at 37 °C in a humidified atmosphere with 5% CO_2_. All cell lines of human origin were authenticated using STR (Single Tandem Repeats) analysis conducted in-house through the Translational Science Biocore shared resource of the University of Wisconsin Carbone Cancer Center. Mycoplasma testing of all cell lines was performed routinely and whenever cultures of new cell stocks were initiated.

Cell culture media and general laboratory supplies and reagents were purchased from ThermoFisher (Waltham MA) unless otherwise stated. Reagents for the Seahorse experiments were obtained from Agilent Technologies. The vendors for the six test compounds were as follows—oryzalin (Aldrich CPR, St.Louis, MO), allylestrenol (TargetMol Chemicals Inc.,Wellesley Hills, MA), esbiothrin/allethrin and coumatetralyl (Sigma-Aldrich, St.Louis, MO), lacidipine (Ambeed, Inc, Arlington Heights, IL), and 2-cyano-*N*-cyclohexyl-3-[5-(4-morpholinyl)-2-furyl]-2-propenamide (CCMP) (Vitas-M Laboratories Ltd, Champaign, IL). All test compounds were purchased through the Aldrich Market Service (Small Molecule Screening Library Service) and except for oryzalin where data was not provided by the vendor, the purity of each compound was > 92%.

### Bioenergetics assays

Oxygen Consumption Rate (OCR): ID-8 (5000/well) were seeded in XFe96 Seahorse Analyzer Culture Plate overnight and next day treated with drugs (oryzalin, allylestrenol, esbiothrin/ allethrin, lacidipine, coumatetralyl, (Z)-2-cyano-*N*-cyclohexyl-3-[5-(4-morpholinyl)-2-furyl]-2-propenamide) (CCMP) at concentrations between 10–40 μM for 45 min. After treatment, cells were washed twice with XF RPMI media containing 1 mM pyruvate, 2 mM glutamine and 10 mM glucose (Agilent Technologies). Culture media was replaced with 180 μl of XF RPMI media and equilibrated for 45 min at 37 °C in a non-CO_2_ incubator. The mitochondrial stress test was performed with injections of oligomycin (1 μM); FCCP (2 and 0.5 μM); and rot/antimycin (1 μM). Oxygen Consumption Rate (OCR) and ATP production were measured and analyzed with Agilent Wave software. The data were normalized to the total protein (μg) in each well.

### Intracellular oxygen radical quantitation

Reactive Oxygen Species (ROS) in the mouse ID8 and human ovarian cancer cell lines OVCAR 5 was determined using the oxygen radical-sensing dye 2,7-Dichlorodihydrofluorescein diacetate (H_2_DCFDA) (Sigma Aldrich) by Flow cytometry. Cells (5 × 10^5^) were washed twice with 1X HBSS, followed by incubation with 10 μM H_2_DCFDA dye in serum free media for 30 min at 37 °C under 5% CO_2_. After incubation, cells were washed twice with 1X HBSS and protected from light. Test compounds at desired concentrations were added to the cells in serum-free media and after 45 min incubation, the cells were analyzed on an Attune (ThermoFisher) spectral flow cytometer. Data was analyzed using FCS Express 7 software.

### Cell viability assays

Cell viability was assessed using the MTT assay as described in our previous studies. OVCAR-5 (2500 cells), ID-8 (1000 cells) were seeded in a flat bottomed—96 well plates and incubated overnight at 37 °C in 5% CO_2_. Control cells were exposed to the relevant concentration of DMSO (vehicle) and test cells were treated with the respective concentration of each of the test compounds. The treatment was performed for 72 h. Following incubation, the MTT reagent (0.5 mg/ml) was added to each well and incubated for 3 h at 37 °C. DMSO (100 µl) was added to each well to dissolve the formazan crystals. Plate was mixed gently for 4–5 min and the optical density of solution in each well was determined using a microplate reader at 560 nm.

Cell viability was also tested by flow cytometry. Cell lines treated with the test drugs were harvested from the culture plates and the dead cell indicator DAPI (2 μl of 5 µg/ml stock in FACS buffer) was added to the cell suspension (100 µl). The cells were analyzed by flow cytometry on the Attune (ThermoFisher) cytometry and the data was analyzed using FCS Express 7 software.

## Supplementary Information

Additional file 1. ETC-linkage terms applied to AID “name”, “title”, or “abstract”. List of terms for a secondary filter consisting of 39 positive terms (involving electron transport in mitochondria) and 2 negative terms (related to the photosynthesis pathway).

Additional file 2. Structures of medoids of each compound cluster. Structures of each of the medoid that are the central compounds of each cluster identified to contain potential OXPHOS inhibitors.

Additional file 3. **A**–**F** UMAP projections of OXPHOS inhibitor candidates. Different 2D UMAP projections of the 2-D chemical space based on the PC_OXPHOS compound set. A-C show the full, inactive, and active compound distributions as KDE represented by contour maps, respectively. In A, points are overlayed for active compounds. Colored points represent different clusters exceeding 2 molecules. Remaining active molecules are shown as grey points. Projections D-F show difference maps derived by subtracting the KDEs of the active and inactive points. Positive values (red) show regions of relatively high OXPHOS-active density. Negative values (blue) show relatively low density for OXPHOS actives. Medoids from active chemical clusters of size 3 or greater are overlayed as labeled points. In D, the OXPHOS-active oxygen-rich saturated polycycles are highlighted. In E, active nitrogenous heterocycles are labeled. Map F shows some miscellaneous OXPHOS actives that do not fit into the latter categories.

Additional file 4. Validation of top performing machine learning models. Model hyperparameters were explored in 5-fold cross validation on 85% of the data and the top performing model from each combination of classifier type and input representation was evaluated.

Additional file 5. Compounds used in training set. List of 51 documented OXPHOS inhibitors and how they score relative to the test set in both RFC and SVC models. 44 of these documented inhibitors were not in the original training set and therefore serve as an additional test set for the models. True labels and classification labels are also provided in addition to model probability scores.

Additional file 6. Feature importance correlations between methods. MDI analysis of the descriptor- and fingerprint-based RF models reveals key features, which appear highly correlated with the features’ absolute mean divergences between active and inactive populations with respect to descriptors and fingerprint bits.

Additional file 7. **A**–**C**. Important ECFP6 bits based on feature permutation analysis of chemical fingerprints in our RFC model. Important ECFP6 bits based on feature permutation analysis of chemical fingerprints in our RFC model. Bits (A) 694, (B) 1917, and (C) 1152 correspond to methine carbons, generic carbonyls, and generic secondary amines, respectively. The red highlighted atoms and bonds correspond to the substructures represented by each bit. Under each compound several parameters are reported. *Bitrnk* is the ranked importance of the bit according to permutation analysis. *Bit* is the bit index within the ECFP6 fingerprint (length=2048). *Scr* is the score observed for the compound by our top performing RFC. *Scr_rank* is the percentile ranking of the score for the compound. *CID* is the PubChem Compound Identifier. *Label* is the ground truth label for the compound (1=active, 0=inactive).

Additional file 8. RDKit Molecular Descriptor Definitions. Descriptors are defined for property-type and fragment-type features on separate sheets. Where available, a reference is provided for the property-type feature origins.

## Data Availability

The original data downloaded from PubChem and ultimate pipeline-processed datasets are available on Zenodo (10.5281/zenodo.11003006). Python scripts with documentation for running the pipeline, generating manuscript figures, and building/evaluating machine learning classifiers are available on GitHub [https://github.com/SpencerEricksen/PubChem_DataMining_OXPHOS].
