# Supplementary material for: Data mining of PubChem bioassay records reveals diverse OXPHOS inhibitory chemotypes as potential therapeutic agents against ovarian cancer
Source: J Cheminform. 2024 Oct 7;16:112. doi: 10.1186/s13321-024-00906-0 (PMC11460086; doi:10.1186/s13321-024-00906-0)
Supplement: Supplementary file 1 — Additional file 1. ETC-linkage terms applied to AID “name”, “title”, or “abstract”. List of terms for a secondary filter consisting of 39 positive terms (involving electron transport in mitochondria) and 2 negative terms (related to the photosynthesis pathway). [file 13321_2024_906_MOESM1_ESM.docx]

| **Additional File 1. ETC-linkage terms applied to AID “name”, “title”, or “abstract”** | | | | |
| --- | --- | --- | --- | --- |
| **Positive terms** | | |  | **Negative terms** |
| mitochondrial | hypoxia | complex I |  | chloroplast |
| mitochondria | NAD1 | complex II |  | thylakoid |
| ROS | bc1 complex | complex III |  |  |
| electron transport | b-c1 complex | UQCR |  |  |
| respiration | bc-1 complex | complex IV |  |  |
| respiratory chain | cytochrome c | complex V |  |  |
| ETC | cytochrome bc1 | NADH-CoQ reductase |  |  |
| OXPHOS | cytochrome b-c1 | NADH dehydrogenase |  |  |
| NADH oxidase | cytochrome bc-1 | succinate dehydrogenase |  |  |
| ubiquinone | ATP synthase | succinate-CoQ reductase |  |  |
| oxidoreductase | oxygen consumption | CoQH2 |  |  |
| ataxia | superoxide | CoQ |  |  |
|  | radical | AA3 |  |  |
|  |  |  |  |  |
|  |  |  |  |  |
|  |  |  |  |  |
