# Supplementary material for: Data mining of PubChem bioassay records reveals diverse OXPHOS inhibitory chemotypes as potential therapeutic agents against ovarian cancer
Source: J Cheminform. 2024 Oct 7;16:112. doi: 10.1186/s13321-024-00906-0 (PMC11460086; doi:10.1186/s13321-024-00906-0)
Supplement: Supplementary file 3 — Additional file 3. A–F UMAP projections of OXPHOS inhibitor candidates. Different 2D UMAP projections of the 2-D chemical space based on the PC_OXPHOS compound set. A-C show the full, inactive, and active compound distributions as KDE represented by contour maps, respectively. In A, points are overlayed for active compounds. Colored points represent different clusters exceeding 2 molecules. Remaining active molecules are shown as grey points. Projections D-F show difference maps derived by subtracting the KDEs of the active and inactive points. Positive values (red) show regions of relatively high OXPHOS-active density. Negative values (blue) show relatively low density for OXPHOS actives. Medoids from active chemical clusters of size 3 or greater are overlayed as labeled points. In D, the OXPHOS-active oxygen-rich saturated polycycles are highlighted. In E, active nitrogenous heterocycles are labeled. Map F shows some miscellaneous OXPHOS actives that do not fit into the latter categories. [file 13321_2024_906_MOESM3_ESM.pptx]

## Slide 1
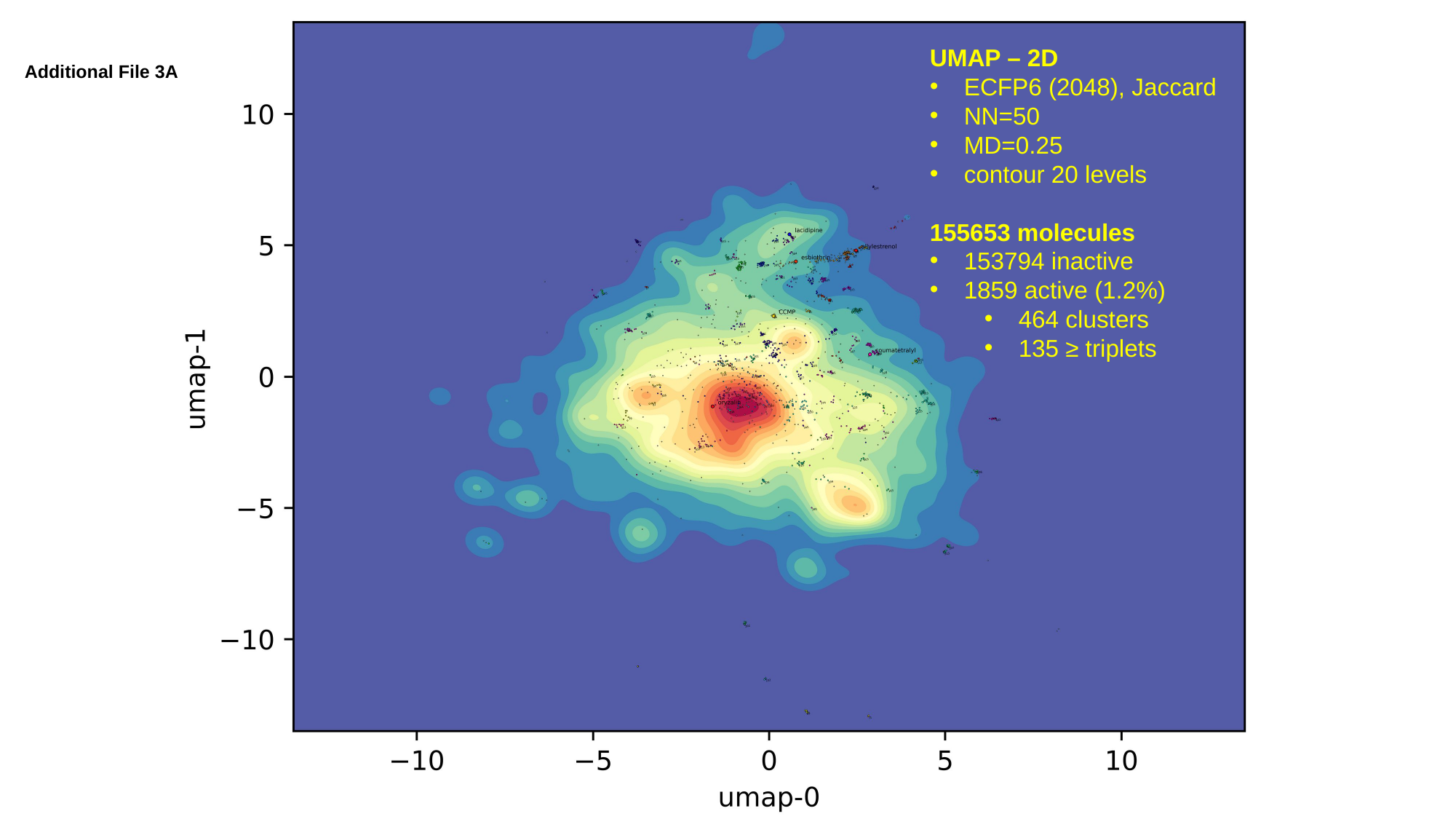

UMAP – 2D
ECFP6 (2048), Jaccard
NN=50
MD=0.25
contour 20 levels
155653 molecules
153794 inactive
1859 active (1.2%)
464 clusters
135 ≥ triplets
Additional File 3A

## Slide 2
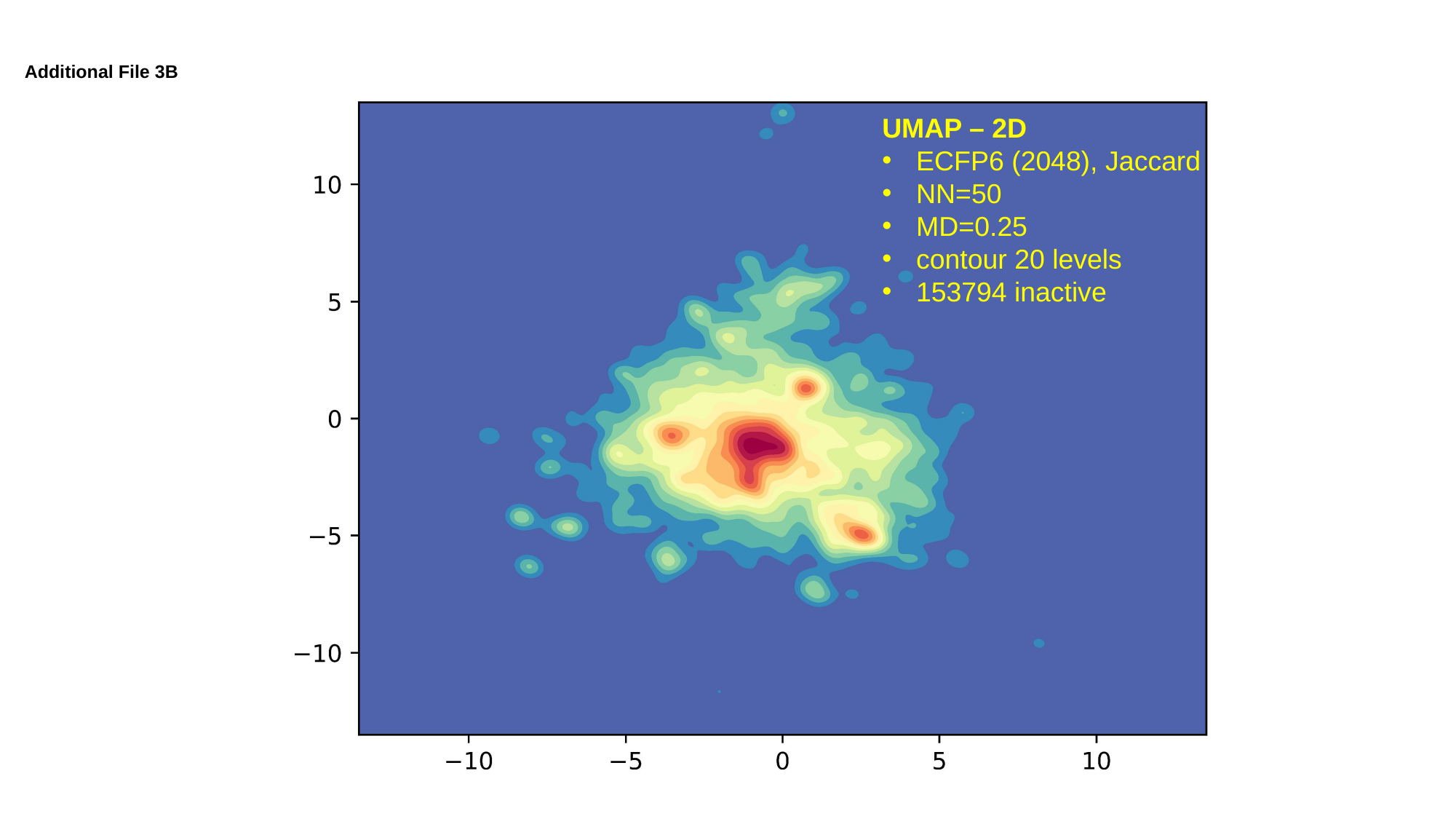

UMAP – 2D
ECFP6 (2048), Jaccard
NN=50
MD=0.25
contour 20 levels
153794 inactive
Additional File 3B

## Slide 3
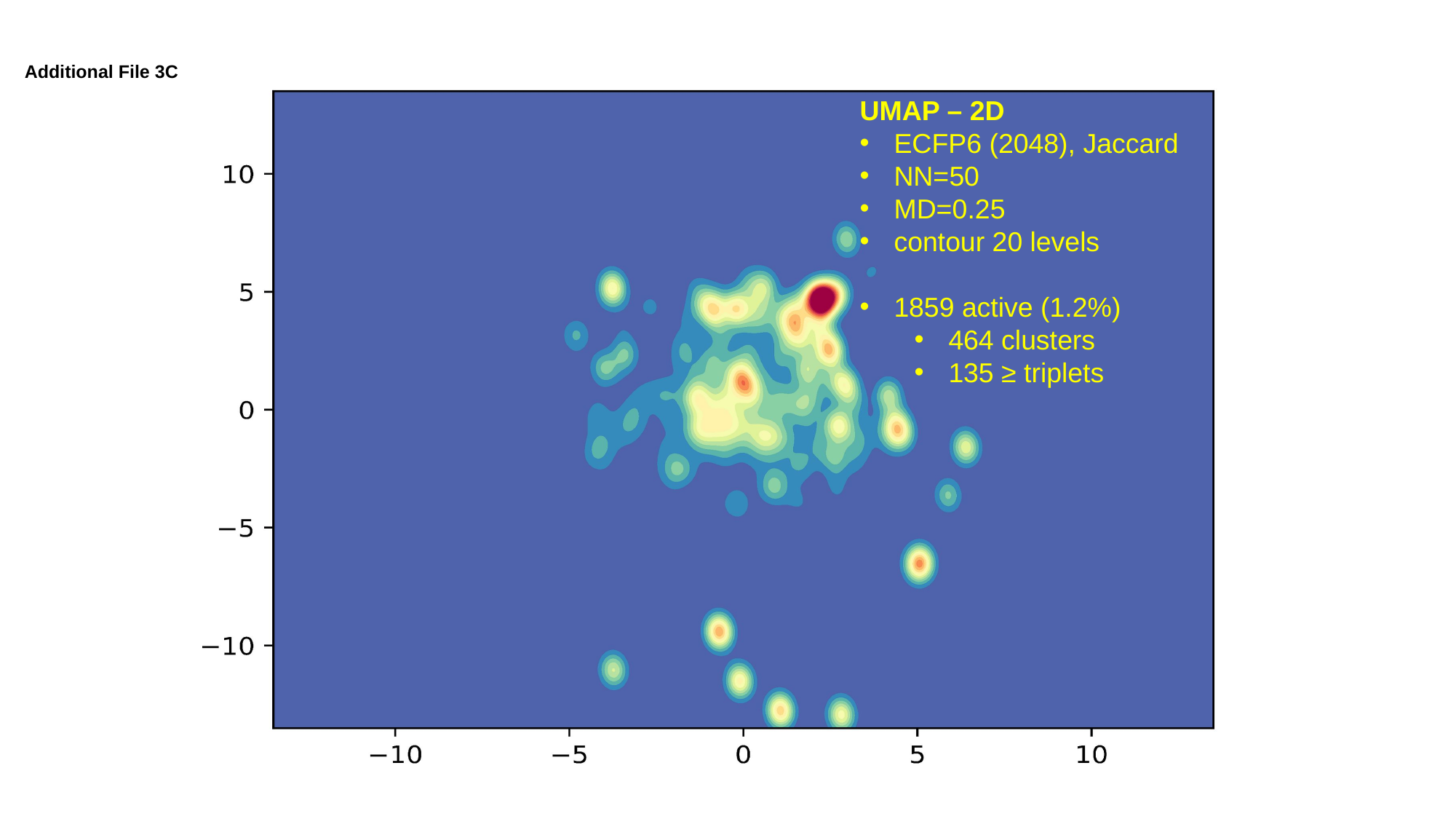

UMAP – 2D
ECFP6 (2048), Jaccard
NN=50
MD=0.25
contour 20 levels
1859 active (1.2%)
464 clusters
135 ≥ triplets
Additional File 3C

## Slide 4
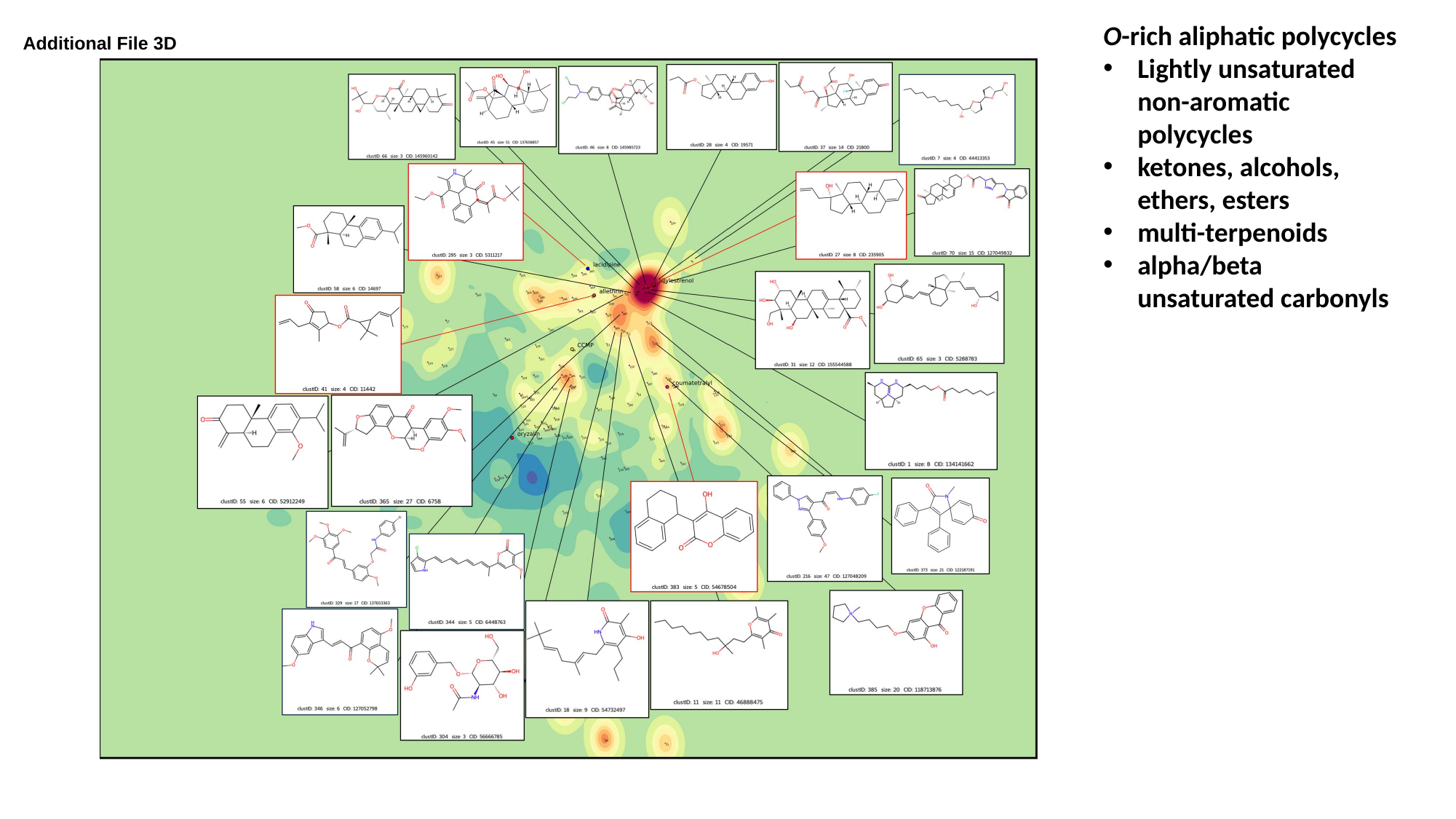

O-rich aliphatic polycycles
Lightly unsaturated non-aromatic polycycles
ketones, alcohols, ethers, esters
multi-terpenoids
alpha/beta unsaturated carbonyls
Additional File 3D

## Slide 5
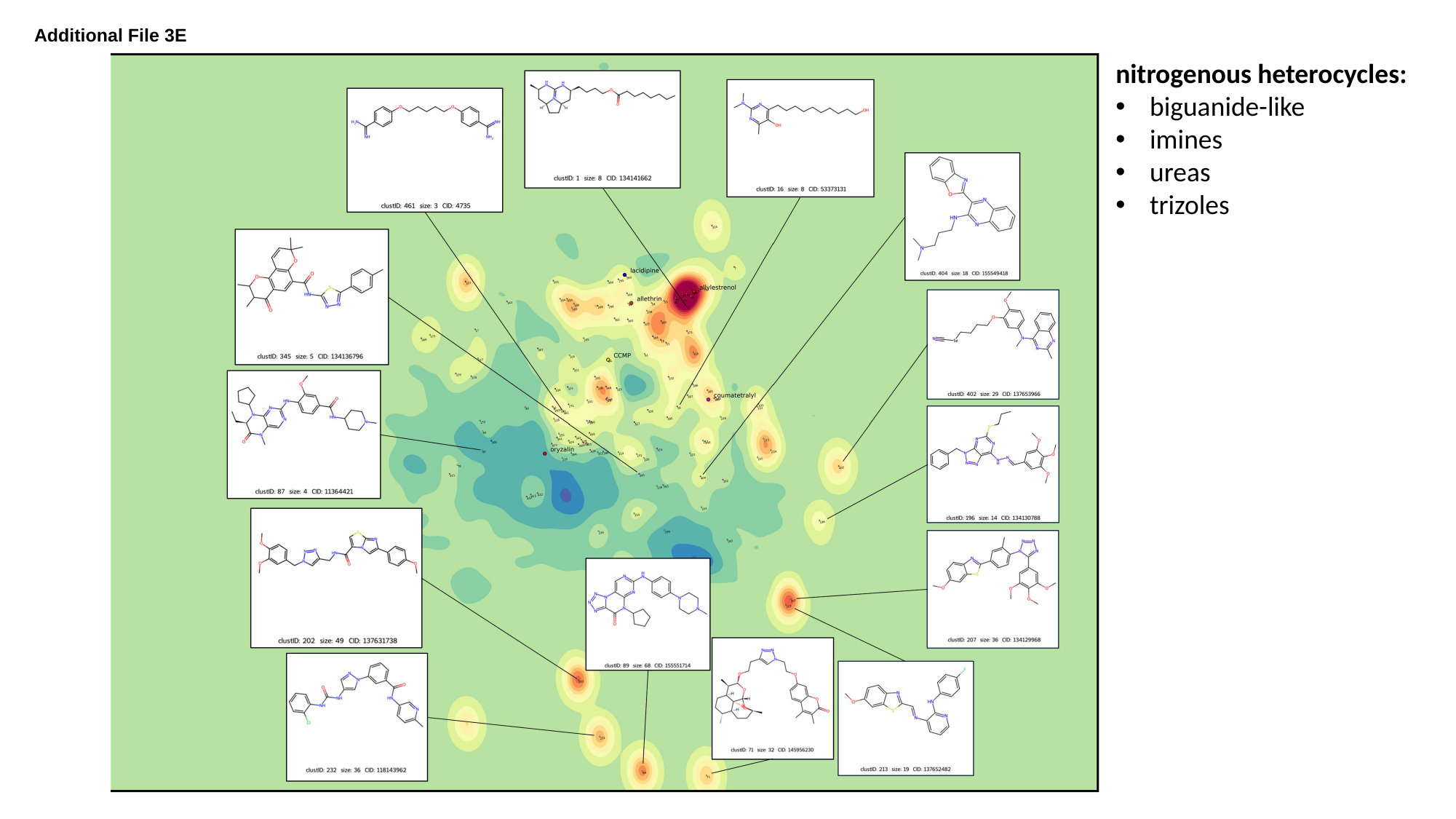

Additional File 3E
nitrogenous heterocycles:
biguanide-like
imines
ureas
trizoles

## Slide 6
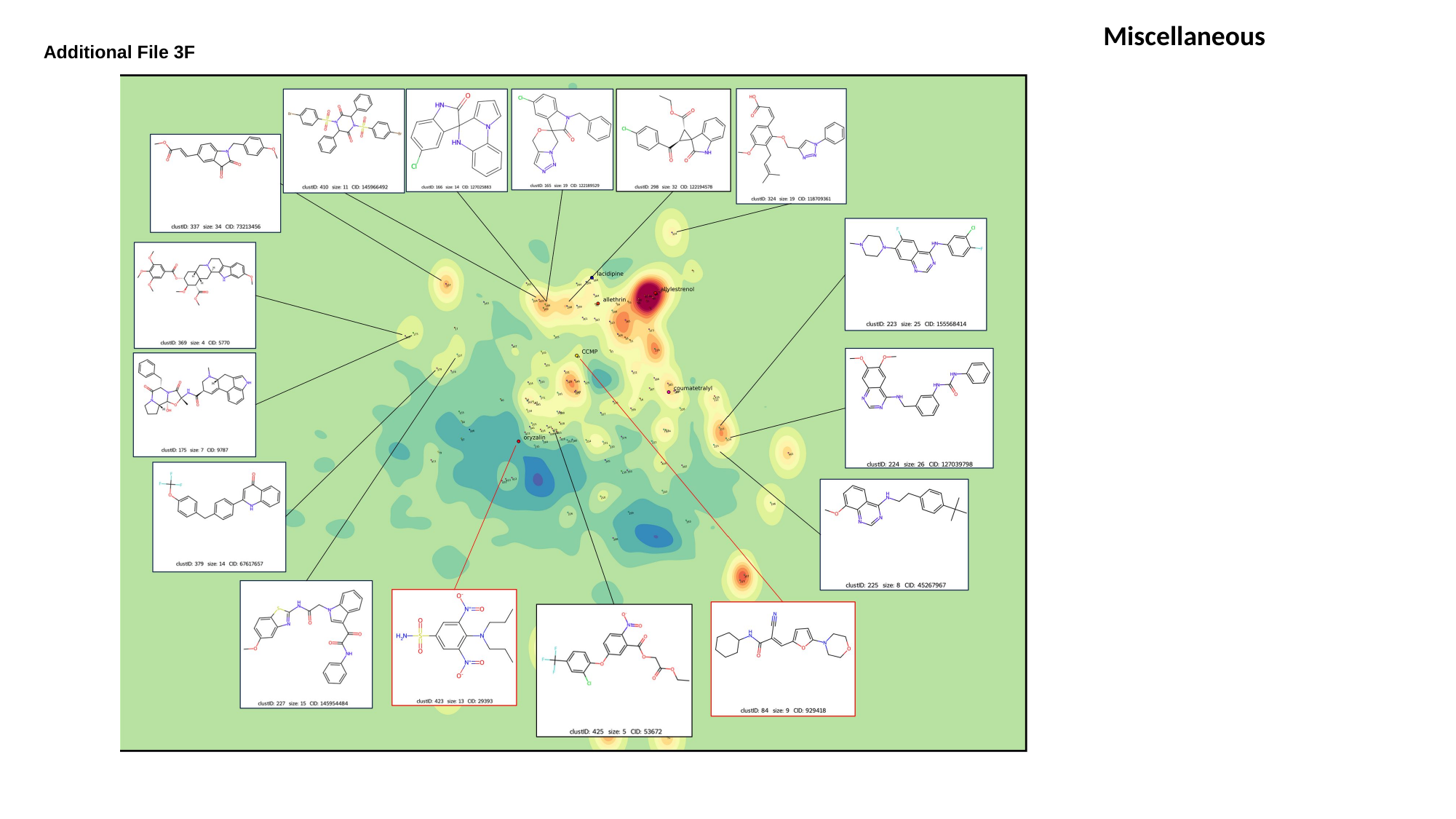

Miscellaneous
Additional File 3F
