# Supplementary material for: Data mining of PubChem bioassay records reveals diverse OXPHOS inhibitory chemotypes as potential therapeutic agents against ovarian cancer
Source: J Cheminform. 2024 Oct 7;16:112. doi: 10.1186/s13321-024-00906-0 (PMC11460086; doi:10.1186/s13321-024-00906-0)
Supplement: Supplementary file 6 — Additional file 6. Feature importance correlations between methods. MDI analysis of the descriptor- and fingerprint-based RF models reveals key features, which appear highly correlated with the features’ absolute mean divergences between active and inactive populations with respect to descriptors and fingerprint bits. [file 13321_2024_906_MOESM6_ESM.docx]

| **Additional File 6. Feature Importance Correlations Between Methods** | | | | | |
| --- | --- | --- | --- | --- | --- |
|  | **Descriptors** | | **Fingerprints** | |  |
|  | MDI | Perm | MDI | Perm |  |
| Div | 0.450 | 0.005 | 0.908 | 0.358 |  |
| MDI |  | 0.562 |  | 0.351 |  |
