# Supplementary material for: Data mining of PubChem bioassay records reveals diverse OXPHOS inhibitory chemotypes as potential therapeutic agents against ovarian cancer
Source: J Cheminform. 2024 Oct 7;16:112. doi: 10.1186/s13321-024-00906-0 (PMC11460086; doi:10.1186/s13321-024-00906-0)
Supplement: Supplementary file 7 — Additional file 7. A–C. Important ECFP6 bits based on feature permutation analysis of chemical fingerprints in our RFC model. Important ECFP6 bits based on feature permutation analysis of chemical fingerprints in our RFC model. Bits (A) 694, (B) 1917, and (C) 1152 correspond to methine carbons, generic carbonyls, and generic secondary amines, respectively. The red highlighted atoms and bonds correspond to the substructures represented by each bit. Under each compound several parameters are reported. Bitrnk is the ranked importance of the bit according to permutation analysis. Bit is the bit index within the ECFP6 fingerprint (length=2048). Scr is the score observed for the compound by our top performing RFC. Scr_rank is the percentile ranking of the score for the compound. CID is the PubChem Compound Identifier. Label is the ground truth label for the compound (1=active, 0=inactive). [file 13321_2024_906_MOESM7_ESM.pptx]

## Slide 1
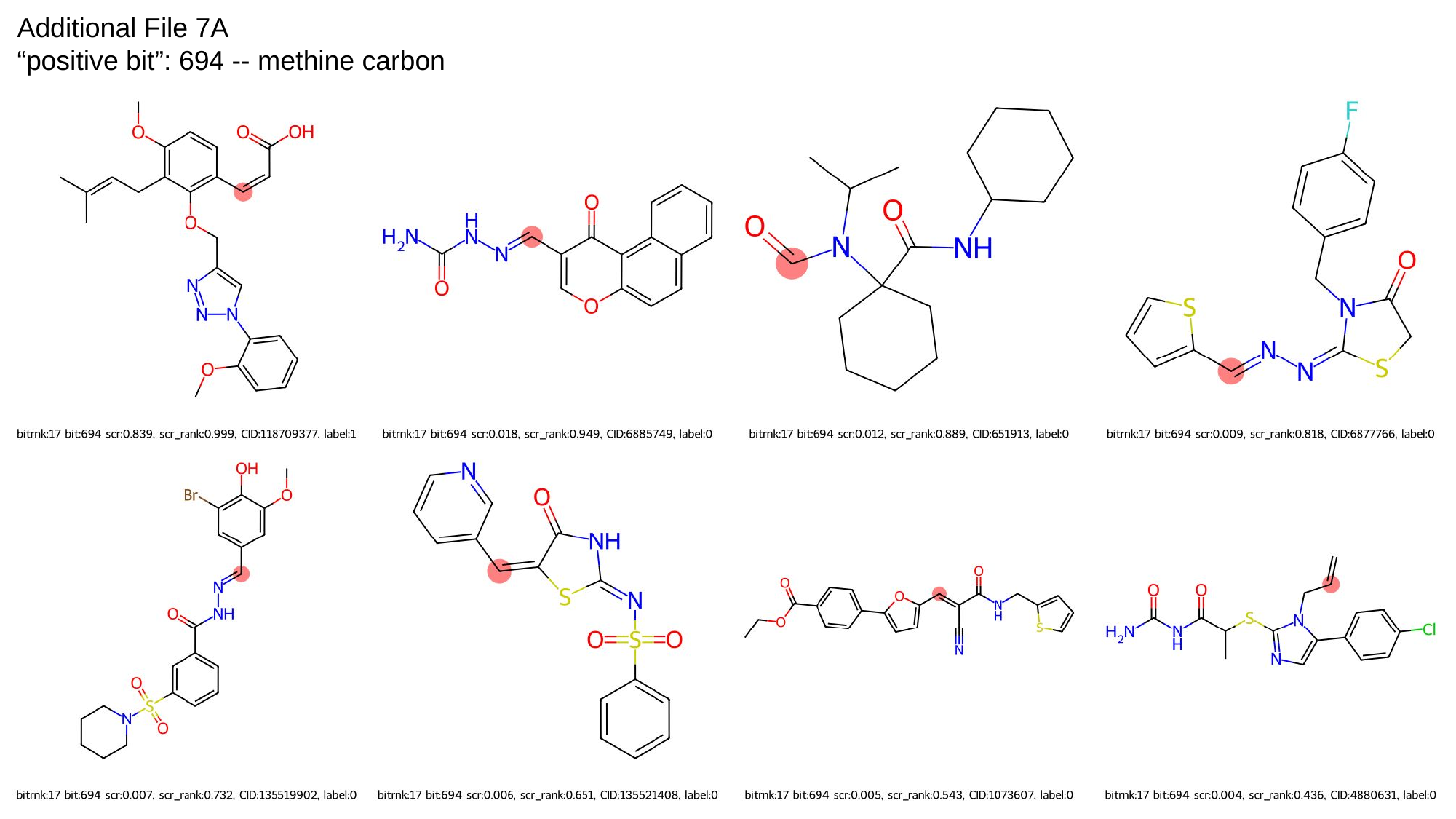

Additional File 7A
“positive bit”: 694 -- methine carbon

## Slide 2
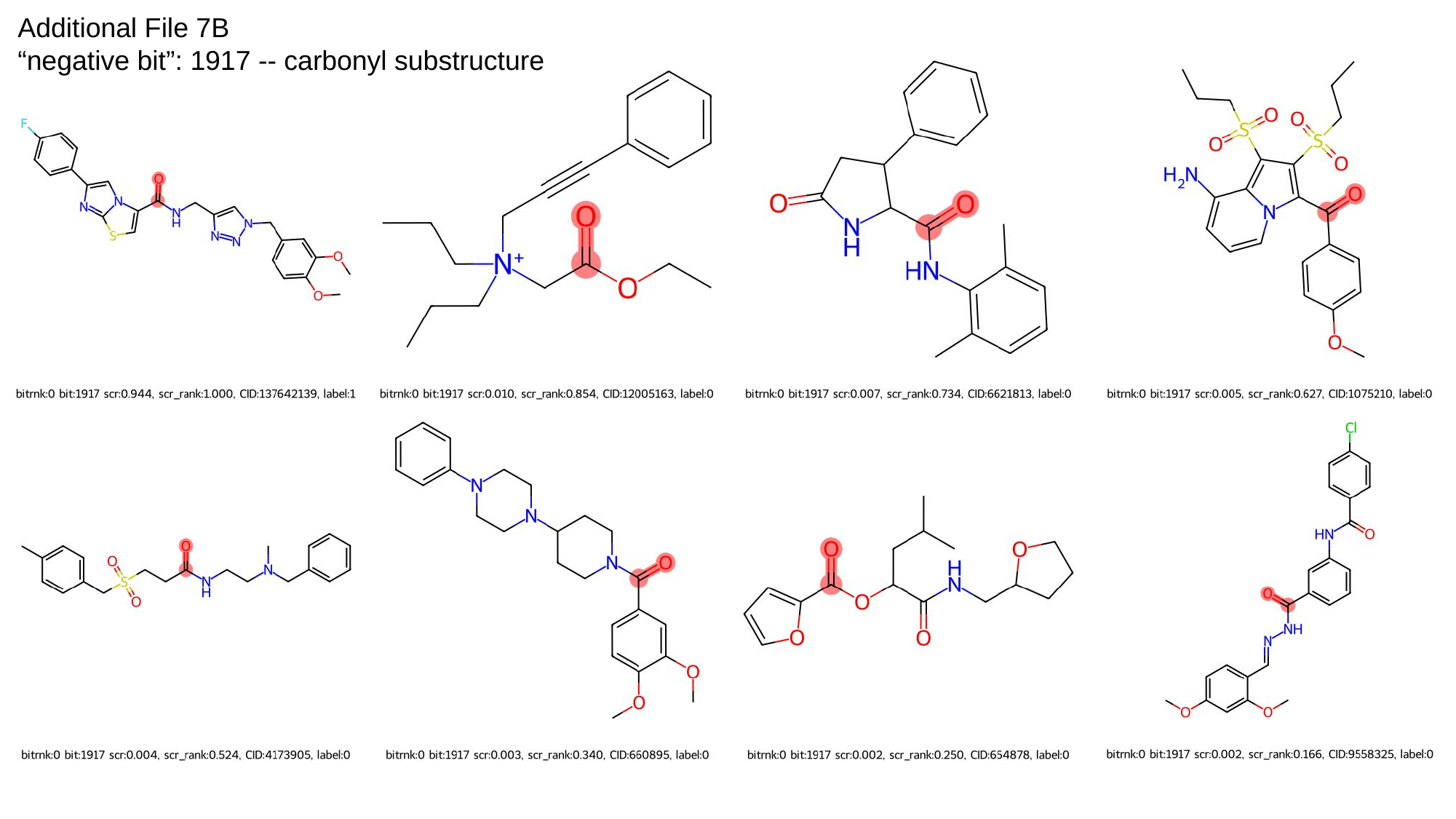

Additional File 7B
“negative bit”: 1917 -- carbonyl substructure

## Slide 3
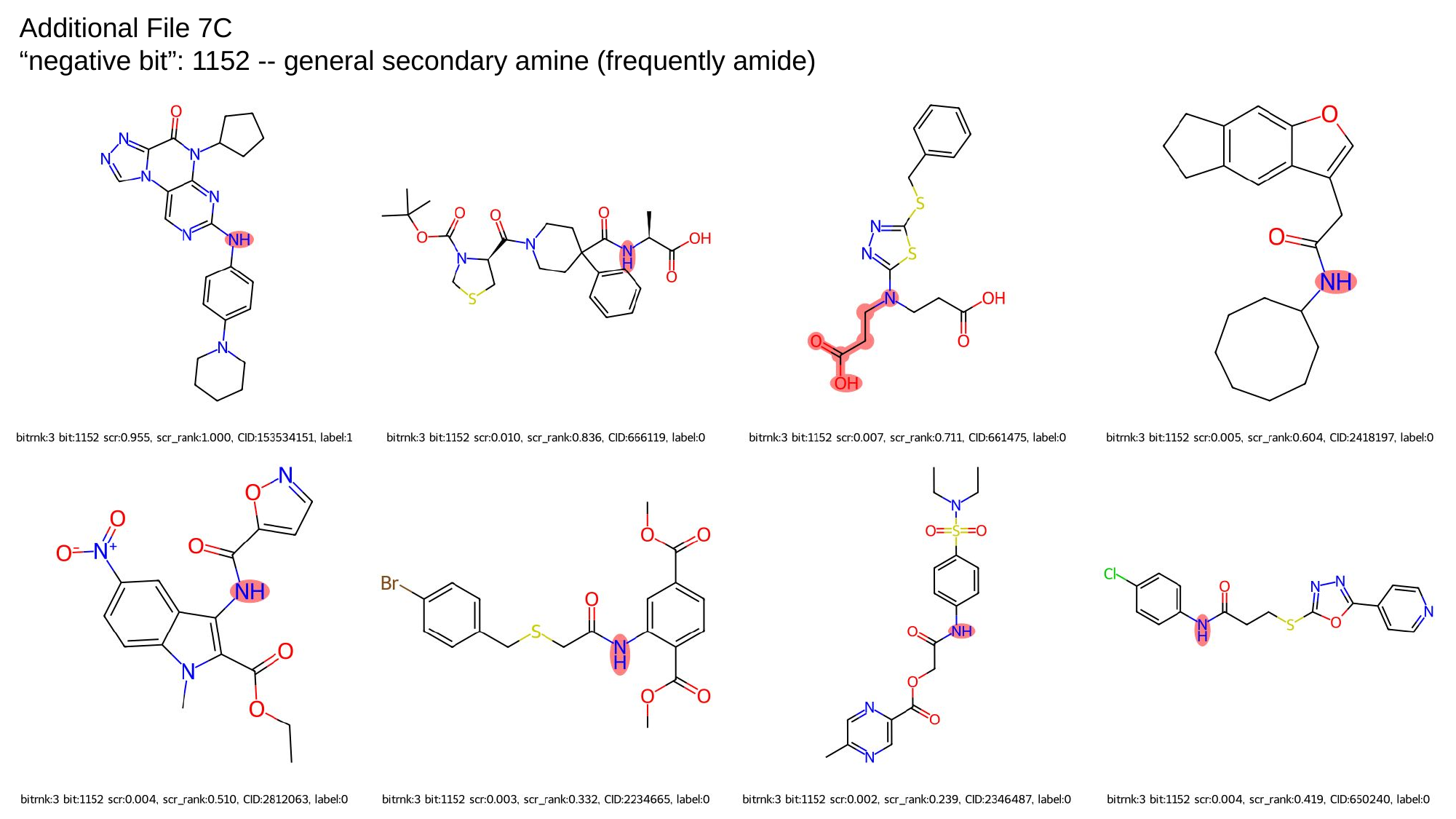

Additional File 7C
“negative bit”: 1152 -- general secondary amine (frequently amide)
